# Activity-Based Probes for Studying the Activity of Flavin-Dependent Oxidases and for the Protein Target Profiling of Monoamine Oxidase Inhibitors**

**DOI:** 10.1002/anie.201201955

**Published:** 2012-06-11

**Authors:** Joanna M Krysiak, Johannes Kreuzer, Peter Macheroux, Albin Hermetter, Stephan A Sieber, Rolf Breinbauer, Dedicated to Professor Herbert Waldmann on the occasion of his 55th birthday

**Affiliations:** Institute of Organic Chemistry, Graz University of TechnologyStremayrgasse 9, 8010 Graz (Austria); Center for Integrated Protein Science CIPSM, Department of Chemistry, Institute of Advanced Studies IAS, Technische Universität MünchenLichtenbergstraße 4, 85747 Garching (Germany); Institute of Biochemistry, Graz University of TechnologyPetersgasse 12, 8010 Graz (Austria)

**Keywords:** activity-based probes, deprenyl, flavin cofactor, monoamine oxidases, proteomics

Activity-based protein profiling (ABPP) has become a powerful chemical proteomic technology allowing the dissection of complex ligand–protein interactions in their native cellular environment.[1] The application of small-molecule activity-based probes to interrogate enzyme activity on the cell level has led to the identification and functional characterization of proteins involved in cancer,[2] signaling pathways,[3] microbial pathogenesis and virulence,[4] host–virus interactions,[5] and other biological processes. However, up to now most ABPP studies have aimed at enzyme classes with well-established catalytic mechanisms and nucleophilic active-site residues participating in the formation of a covalent bond to activity-based probes (e.g. serine hydrolases,[6] cysteine[7] and threonine proteases[8]). Thus, one important challenge in ABPP is expanding the pool of probe molecules to enzyme classes with more complex catalytic activities such as kinases,[3, 9] transferases,[10] and oxidoreductases[11] to extend the proteome coverage. Here, we introduce unprecedented activity-based probes for an important group of oxidoreductases, namely flavin-dependent oxidases.

Flavin-dependent enzymes catalyze a diverse set of reactions encompassing oxidations, monooxygenations, dehydrogenations, reductions, and halogenations, making them indispensable for many cellular processes.[12] Among them, flavin-dependent oxidases represent a complex subgroup that oxidize a broad spectrum of molecules by the employment of molecular oxygen as an electron acceptor.[13] Their intrinsic structural diversity, multiplicity of accepted substrates, and lack of conserved residues in the active site make them elusive to functional annotation by established genomic, structural, and proteomic analyses.[14] In contrast, ABPP could serve as a powerful and simple alternative for global profiling of these enzymes. We envisioned that selective activity-based probes could be built on the simple principle of the binding affinity of the oxidatively activated probes towards the flavin cofactor, the only common and intrinsic feature of flavin-dependent oxidases.[15]

We present here the development and biological evaluation of a novel chemoproteomic strategy dedicated to flavin-dependent oxidases which involves a “tag-free” approach for in situ enzyme labeling within intact cells. Subsequent cell lysis followed by click chemistry[16] results in the attachment of the fluorescent tag which serves in the visualization of enzyme activities by gel electrophoresis and fluorescence scanning. A LC–MS-based platform finally reveals the identity of labeled enzymes (Figure 1 A).

**Figure 1:**
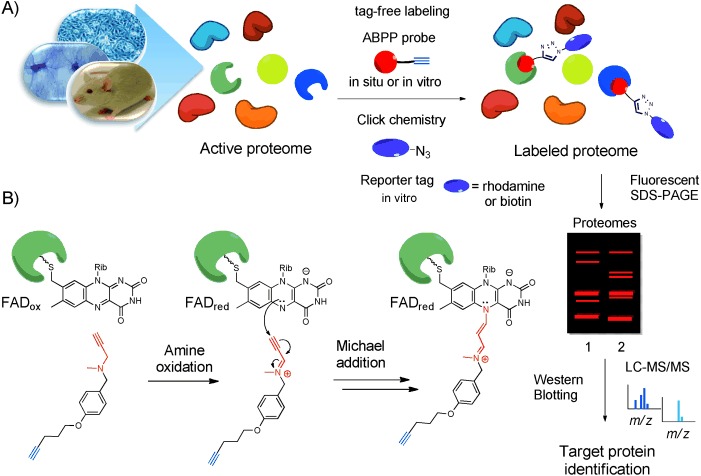
A) Identification of a target protein by the use of ABPP. B) Labeling of activity-based probes dedicated to monoamine oxidases (FAD-dependent oxidases).

The designed ABPP methodology was examined using a monoamine oxidase enzyme, a representative example of flavin-dependent oxidases, to validate the new labeling mechanism on a well-known target. Monoamine oxidases[17] (MAO, EC 1.4.3.4) are flavin adenine dinucleotide (FAD)-containing enzymes, localized in the mitochondrial outer membrane, which catalyze the oxidative deamination of several important neurotransmitters in the central nervous system (CNS), including serotonin, norepinephrine, and dopamine as well as xenobiotic amines. In humans, monoamine oxidases exist in two isoforms designated MAO A and MAO B,[18] which are encoded by two distinct genes[19] on the X chromosome and display unique substrate selectivities and inhibitor sensitivities[18] although they share a high level of sequence identity (70 %).[19] The elucidation of the crystal structures of isozymes MAO A[20] and MAO B[21] provided detailed insight into structural differences in their active sites accounting for distinct substrate and inhibitor specificities.[20, 22] Moreover, it confirmed the covalent attachment[23] of the isoalloxazine ring of the FAD cofactor through an 8α-(*S*-cysteinyl) linkage to a cysteine residue.[24] On account of their involvement in catabolism and the regulation of neurotransmitters, monoamine oxidases play an essential role in normal brain development and function. It was found that mutations in the MAO A gene are implicated in manifestations of aggressive behavior[25] while age-related increases of expression levels of MAO A in the heart[26] and MAO B in neuronal tissue[27] have been associated with the development of cardiovascular[28] and neurodegenerative disorders,[29] respectively. Hence, MAO inhibitors are clinically used for the treatment of depression, Parkinson’s disease, anxiety disorders, and other mental diseases.[30]

We designed and synthesized a small set of novel activity probes (Figure 2 A) based on the structure of the known irreversible MAO inhibitors pargyline and deprenyl (Figure 2 B). These inhibitors have been used in the functional and biochemical characterization of monoamine oxidases and are useful drugs in clinical applications.[31, 32] Both inhibitors feature an *N*-propargylamine group which is essentially involved in irreversible enzyme inhibition and the formation of a stable covalent adduct, whose existence has been unambiguously proven by crystal structures of MAO with acetylenic inhibitors such as deprenyl, clorgyline, and rasagiline.[20, 33] In the initial step of this inhibition mechanism, the FAD cofactor catalyzes the oxidation of the amine group to an iminium cation producing a reactive Michael acceptor, which can be nucleophilically attacked by the N(5) atom of the isoalloxazine ring leading to the formation of the covalent adduct. We anticipated that activity-based probes built on these inhibitors will employ the same labeling mechanism (Figure 1 B).

**Figure 2:**
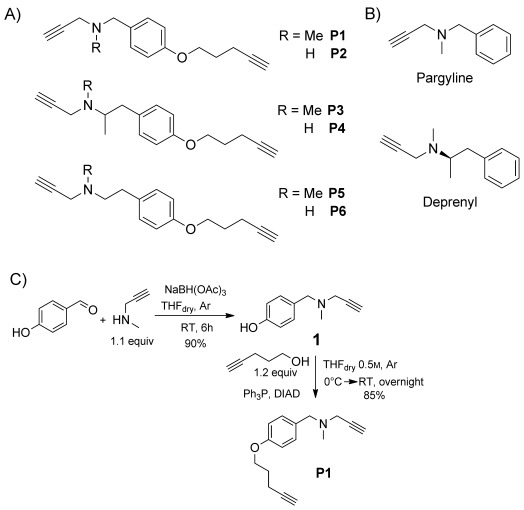
Design and synthesis of ABP probes for monoamine oxidases (MAO). A) Structures of sets of probes inspired by irreversible monoamine oxidase inhibitors. B) Structures of irreversible MAO inhibitors: pargyline (unspecific), deprenyl (specific for MAO B). C) Synthesis of probe **P1**.

The straightforward synthesis of the ABPP probes started with a reductive amination to introduce the desired propargylamine group. Mitsunobu reaction with 4-pentyn-1-ol allowed the attachment of an alkyl linker carrying an alkyne group under very mild conditions to furnish the appropriate probes (Figure 2 C). Structurally diverse products were prepared through the use of different substitution patterns at the amino group and by the various lengths and structures of the carbon skeleton between the reactive group and arene ring.

To test whether the incorporation of an alkyne handle into the pargyline and deprenyl changes their MAO inhibition potency, we determined IC_50_ values of the synthesized probes in a continuous Amplex Red/peroxidase-coupled assay and compared them to the parent inhibitors (Figure S1 in the Supporting Information). Fortunately, the structural modifications introduced to the probes did not affect significantly the inhibition of either isozymes. Probe **P3**, a racemic version of deprenyl equipped with an alkyne, showed a decrease in inhibition of MAO B, whereas probe **P1**, based on pargyline, showed an improvement of its IC_50_ value, compared to that of the parent inhibitor.

Having shown that the designed probes meet the requirement of inhibition, we set out to study their use in vitro using *Pichia pastoris* membrane preparations overexpressing human monoamine oxidase. Recombinant human MAO preparations were incubated with the corresponding probe for 1 h at room temperature followed by the attachment of TAMRA-azide tag (Figure S2 A in the Supporting Information) by copper(I)-catalyzed alkyne–azide cycloaddition (click chemistry, CC).[16] SDS-PAGE and in-gel fluorescence scanning were employed to detect labeling events (Figure 3).

**Figure 3:**
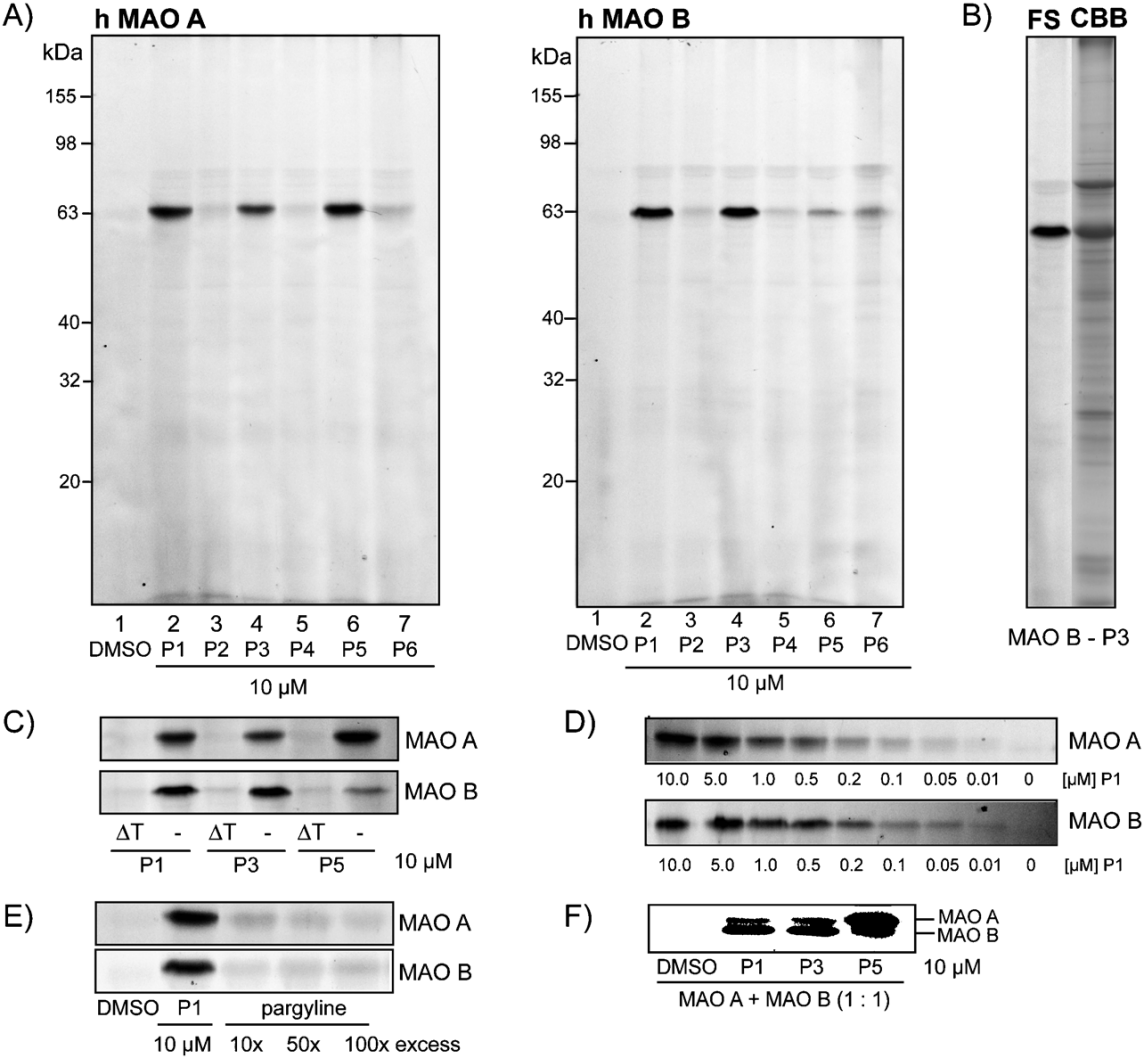
Fluorescent SDS-PAGE analysis of the labeling of monoamine oxidases A and B in vitro. A) Screening of alkyne probes **P1**–**P6** with MAO A and MAO B. B) Comparison of fluorescence scanning (FS) and Coomassie Brilliant Blue (CBB) staining of the labeling of MAO B by the probe **P3**. C) Labeling of MAO A and MAO B after heat denaturation (6 min at 96 °C) (right) and without heat denaturation (left) of the enzyme. D) Concentration-dependent labeling of MAO A and MAO B by probe **P1**. E) Competitive labeling of MAO A and MAO B with pargyline and probe **P1**. F) Labeling of mixture of both MAO isoforms with probes **P1**, **P3**, and **P5**.

In these initial proof-of-concept experiments we could demonstrate that both isoforms of MAO can be efficiently labeled as a main target by probes **P1**, **P3**, and **P5** (Figure 3 A and B) with slightly different isoform preferences (Figure 3 F) at concentrations as low as 100 nm (Figure 3 D). Interestingly, all potent probes identified in the screening (**P1**, **P3**, **P5**) include methyl-substituted tertiary amines, indicating that this feature might be important for efficient labeling. The structural analogues without a methyl substituent at the amino group (**P2**, **P4**, **P6**) exhibit very weak labeling, suggesting that a secondary amino group decreases the interactions between a probe and the protein or has unfavorable redox behavior. Importantly, labeling is completely abolished when the protein is deactivated by heat denaturation prior to incubation with the probe (Figure 3 C), suggesting that labeling occurs only with the active enzyme in a specific manner. In competition experiments we could show that our ABPP probes compete with MAO-specific inhibitors for the same binding site (flavin cofactor) in the enzyme active site since pargyline is able to efficiently block the enzyme labeling by probe **P1** (Figure 3 E). Collectively, these results demonstrate that the developed ABPP system can serve as an effective chemical tool for profiling activity of both isoforms of MAO in vitro. These valuable results prompted us to evaluate further the potency and selectivity of the best probes **P1** and **P3** in profiling the activity of monoamine oxidases in far more complex biological samples. Additionally, we were interested in determining whether these probes are able to target other flavin-dependent enzymes.

First, we evaluated the general labeling properties of probes **P1** and **P3** with different mouse tissue homogenates (heart, lung, brain). Interestingly, the only specific bands were noticeable in the insoluble fraction of mouse brain lysate (Figure S3 in the Supporting Information). Probe **P1**, based on the unspecific MAO inhibitor pargyline, labeled two proteins in the range of 60 kDa, whereas probe **P3**, based on the MAO B specific inhibitor deprenyl, labeled only one target, which was identical to the lower band labeled by probe **P1**. The labeling was dose-dependent and was still observable at a concentration of 100 nm (Figure S3 B in the Supporting Information). Interestingly and importantly, the molecular weights of the labeled bands matched up with those of MAO A (higher band) and MAO B (lower band) (Figure S3 C), and the labeling was completely abolished when the lysate was first incubated with excess of MAO inhibitors (Figure S3 D).

Encouraged by these initial results, we rationalized that since pargyline and deprenyl are applied in the study and treatment of CNS disorders (deprenyl is used as an antiparkinson drug[32]), a human brain cancer cell line (designated RAEW) isolated from a patient who was suffering from a glioblastoma multiforme (GBM) tumor, would be a suitable system for target validation.[34] Prior to ABPP labeling we determined the cytotoxicity of probes **P1** and **P3** against an eukaryotic cell line (GBM model, DBTRG-05MG) and validated that within the range of concentrations used for ABPP experiments the cells were still viable (Figure S4 in the Supporting Information).

In situ labeling with GBM cells revealed that both probes **P1** and **P3** labeled two bands in the range of 63 kDa as main protein targets; however, the labeling by probe **P3** proved to be much more effective (Figure 4 A). A 50 μm concentration of probe **P3** was sufficient for achieving binding saturation as higher concentrations did not improve the labeling of the much weaker lower band. Importantly, inhibitors of monoamine oxidases outcompeted the labeling by probe **P3**, suggesting that these two proteins are MAO A (60.5 kDa)[35] and MAO B (59.4 kDa).[35] For unequivocal identification of the two probe-bound proteins we performed a quantitative proteomic analysis using a trifunctional reporter tag (biotin-TAMRA-azide, Figure S2 B), which was attached to a probe **P3** under CC conditions to allow visualization, enrichment, and subsequent identification of proteins by mass spectrometry.4e Analysis of peptide fragments employing the SEQUEST algorithm identified MAO A with nearly 40 % protein coverage (upper band on a fluorescent SDS-PAGE). Unfortunately, the enrichment on avidin beads was insufficient for the lower protein band; however, this protein was unambiguously identified as MAO B by Western blot analysis using specific anti-MAO B antibodies (Figure 4 C).

**Figure 4:**
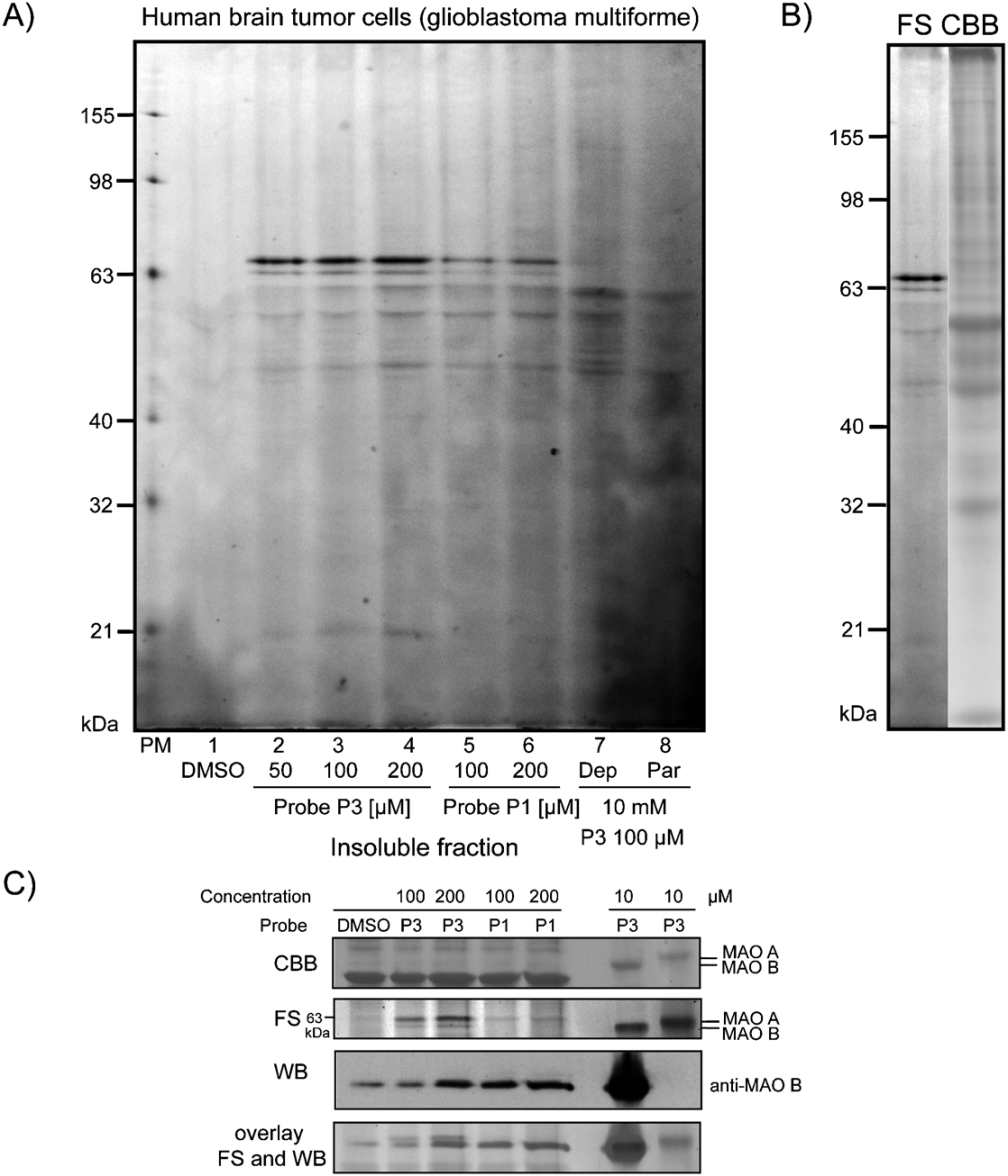
A) In situ ABPP labeling of human brain cancer cells (insoluble fraction, for the whole proteome see Figure S5 in the Supporting Information) with probes **P1** (lanes 5 and 6) and **P3** (lanes 2–4). Competitive labeling with MAO inhibitors deprenyl (Dep) and pargyline (Par) and probe **P3** (lanes 7 and 8). B) Comparison of fluorescence scanning (FS) and Coomassie Brilliant Blue (CBB) staining of labeling by the probe **P3**. C) Identification of lower band by Western blotting (WB) using specific anti-MAO B antibodies.

The results of labeling in live brain cells strikingly demonstrate that the covalently binding inhibitors pargyline and deprenyl act very selectively with MAO A and B but with no other protein targets. This outstanding selectivity is triggered by a unique “suicide” inhibition mechanism that is customized for this enzyme family. This is in contrast to other ABPP studies which identified many off-targets of clinically used covalent drugs.[36]

Probe **P3**, based on the MAO B specific inhibitor deprenyl, showed distinct isozyme preference in in vitro and in situ labeling. In experiments with recombinant proteins, this probe labeled both isoforms of MAO in agreement with studies indicating loss of MAO B specificity at high concentrations.[37] However, **P3** demonstrated higher binding affinity towards MAO B (Figure 3 F), which was also noticeable in mouse brain tissue, in which only the lower protein band, presumably MAO B, was labeled (Figure S3 A). Interestingly, in living human brain tumor cells, this probe was bound preferentially to MAO A. This important result can be explained by the fact that the activity of MAO A is much higher in intact cells than in the purified enzyme, which is known to be particularly unstable at ambient temperatures and loses its activity rapidly.[38] Moreover, one could speculate that the topology of the mitochondrial outer membrane (MOM) of both MAO isozymes can influence the access of the probe to the enzyme. Recent studies[39] demonstrated that MAO A is localized at the cytosolic face in intact rat liver mitochondria and in intact human placental mitochondria,[40] while MAO B resides in the intermembrane space,[39] which can pose some difficulties for the probe in MOM permeability.[41] On the other hand, the weak labeling of MAO B correlates with lower activity of MAO B found in some cultured brain cells;[42] although both MAO isoforms are expressed in human brain at similar levels.[43] Taken together, these results underscore the relevance of in situ studies on enzyme activity, which is apparently not a simple function of enzyme abundance but is tightly regulated by many dynamic processes taking place exclusively in intact living cells.

In conclusion, we have presented the first activity-based probes targeting a flavin-dependent oxidase. We could demonstrate their utility in ABPP studies with both tissues and live cells, particularly in exploring the activity of monoamine oxidases. The unusual labeling mechanism assured outstanding selectivity of the probe molecules which made it possible to study the potential off-target interactions of the clinically used drug deprenyl. We could show that it reacted exclusively with MAO A and B. However, we are convinced that the scope of our novel chemoproteomic approach can be extended by careful fine-tuning of the probe core structure which can result either in higher specificity of the probes or a broader spectrum of the targeted flavin oxidases. Research in this direction is currently underway in our laboratories.

## Supplementary material

Detailed facts of importance to specialist readers are published as ”Supporting Information”. Such documents are peer-reviewed, but not copy-edited or typeset. They are made available as submitted by the authors.
