# Supplementary material for: Activity-Based Probes for Studying the Activity of Flavin-Dependent Oxidases and for the Protein Target Profiling of Monoamine Oxidase Inhibitors
Source: Angew Chem Int Ed Engl. 2012 Jun 11;51(28):7035–40. doi: 10.1002/anie.201201955 (PMC3470703; doi:10.1002/anie.201201955)

Angewandte Chemie
Eine Zeitschrift der Gesellschaft Deutscher Chemiker

Supporting Information

© Wiley-VCH 2012

69451 Weinheim, Germany

Activity-Based Probes for Studying the Activity of Flavin-Dependent Oxidases and for the Protein Target Profiling of Monoamine Oxidase Inhibitors**

Joanna M. Krysiak, Johannes Kreuzer, Peter Macheroux, Albin Hermetter, Stephan A. Sieber, and Rolf Breinbauer**

anie_201201955_sm_miscellaneous_information.pdf

Supporting Information

Experimental Part

Synthesis

General

Chemicals and solvents were purchased from ABCR, Alfa Aesar, Acros Organics, Fisher Scientific or Sigma-Aldrich. All chemicals were of reagent grade or better and used as received. THF was distilled over sodium/benzophenone and stored over 4Å molecular sieves under argon atmosphere. Other solvents were commercially available (dry, stored over molecular sieves and under argon, water <0.005 ppm). Solvents used for flash column chromatography and workup were generally of analytical grade. All experiments were carried out using classical Schlenk techniques under an inert atmosphere of argon or nitrogen. Temperatures were measured externally. Flash column chromatography was performed on silica gel 0.035-0.070 mm, 60Å (Acros Organics). Thin layer chromatography (TLC) was carried out on Merck TLC silica gel 60 F_{254} aluminium sheets and spots were visualized by UV light (λ = 254 nm) or by staining with cerium ammonium molybdate (CAM) solution. ^{1}HNMR and ^{13}C NMR spectra were recorded on a Bruker AVANCE III 300 spectrometer (^{1}H: 300.36 MHz; ^{13}C: 75.53 MHz) or Varian Unity Inova 500 (^{1}H: 499.76 MHz; ^{13}C: 125.66 MHz). Chemical shifts were referenced to the residual proton and carbon signal of the deuterated solvent, respectively (MeOD-d_4: δ = 3.31 ppm (^{1}H), 49.00 ppm (^{13}C), $CDCl_3$: δ = 7.26 ppm (^{1}H), 77.16 ppm (^{13}C)). GC-MS analyses were carried out on an Agilent Technologies 7890A GC system equipped with a 5975C mass selective detector (inert MSD with Triple Axis Detector system, EI, 70 eV) and a J&W GC HP-5MS column (30 m x 0.25 mm x 0.25 µm) at a constant helium flow rate (1.085 mL/min). General gradient temperature method was used (initial temperature: 50 °C for 1 min, linear increase 40°C/min to 300 °C for 5 min, 1 min post-run at 300°C). High resolution mass spectrometry (HRMS) spectra were recorded on Waters GCT Premier Micromass system using a direct inlet (DI) mode, electron impact (EI) ionization

(70 eV) and time of flight (TOF) MS detector in a positive mode. Melting points were measured on Mel-Temp™ melting point apparatus (Electrothermal) and were not corrected.

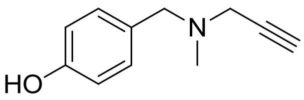

4-((methyl(prop-2-yn-1-yl)amino)methyl)phenol (1)

In an oven dried, evacuated and argon purged 100 mL Schlenk flask 610 mg 4-hydroxybenzaldehyde (5.0 mmol, 1.0 eq) were dissolved in 20 mL dry and degassed THF at RT resulting in a clear yellowish solution. Subsequently, 380 mg *N*-methylpropargylamine (464 µL, 5.5 mmol, 1.1 eq) were added at once resulting in a bright yellow solution. The mixture was stirred at RT for 30 min. Then 1.483 g sodium triacetoxyborohydride (7.0 mmol, 1.4 eq) were added at once. After addition of the reducing agent the mixture became thicker and cloudy. The reaction was monitored by GC-MS and TLC analysis and carried out for 5 h at RT until complete consumption of the aldehyde was indicated. After that time the reaction mixture was quenched with 25 mL saturated $NaHCO_3$. The solution was extracted with ethyl acetate (4 x 20 mL). The combined organic layers were washed with water (2 x 20 mL) and brine (1 x 20 mL) and dried over anhydrous Na_2SO_4. The solvent was removed in *vacuo* and the crude product purified by flash column chromatography (cyclohexane:ethyl acetate = 7:3 (v/v)) furnishing 790 mg (90%) 4-((methyl(prop-2-yn-1-yl)amino)methyl)phenol (**1**) as a pale yellowish solid. ^{1}H NMR (300 MHz, MeOD-d_4) δ: 7.14 (d, *J* = 8.7 Hz, 2H, arom.), 6.74 (d, *J* = 8.7 Hz, 2H, arom.), 3.50 (s, 2H, C**H**$_2$NR$_2$), 3.24 (d, *J* = 2.1 Hz, 2H, C**H**$_2$C≡CH), 2.69 (t, *J* = 2.4 Hz, 1H, CH$_2$C≡C**H**), 2.30 (s, 3H, C**H**$_3$). ^{13}C NMR (75 MHz, MeOD-d_4) δ: 158.06, 131.93, 129.14, 116.09, 78.77, 75.37, 60.20, 44.98, 41.69. GC-MS (EI): t_R = 5.81 min; m/z (%) 175.1 (39) [M^+], 158.2 (2), 148.1 (3), 134.0 (13), 107.0 (100), 82.1 (15), 78.1 (40), 68.1 (45), 52.0 (17). R_f = 0.35 (cyclohexane:ethyl acetate = 1:1 (v/v)), 0.16 (cyclohexane:ethyl acetate = 7:3 (v/v)). m.p. 105-107 °C (pale yellowish solid). HRMS (EI) cald for $C_{11}H_{13}NO$ 175.0997 [M^+], found 175.0998.

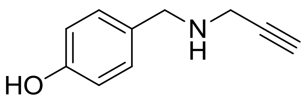

4-((prop-2-yn-1-ylamino)methyl)phenol (2)

In an oven dried, evacuated and argon purged 100 mL Schlenk flask 733 mg 4-hydroxybenzaldehyde (6.0 mmol, 1.0 eq) were dissolved in 36 mL dry and degassed mixture of DCE:THF 5:1 (v/v) at RT forming a clear yellowish solution. Subsequently, a solution of 397 mg propargylamine (461 µL, 7.2 mmol, 1.2 eq) in 1 mL dry DCE was added at once resulting in a bright yellow solution. The mixture was stirred at RT for 30 min. Then 1.780 g sodium triacetoxyborohydride (8.4 mmol, 1.4 eq) were added at once. After addition of the reducing agent the mixture became thicker and cloudy. The reaction was monitored by GC-MS and TLC analysis and carried out overnight for 18 h at RT until nearly complete consumption of the aldehyde was indicated. After that time the reaction mixture was quenched with 20 mL saturated $NaHCO_3$. The solution was extracted with ethyl acetate (3 x 40 mL) and the combined organic layers were washed with water (2 x 50 mL) and brine (1 x 50 mL) and dried over anhydrous Na_2SO_4. The solvent was removed *in vacuo* and the crude product purified by flash column chromatography (cyclohexane:ethyl acetate = 7:3 (v/v) and 1% Et_3N) resulting in 528 mg (55%) 4-((prop-2-yn-1-ylamino)methyl)phenol (**2**) as a dark yellow solid. ^{1}H NMR (500 MHz, MeOD-d_4) δ: 7.15 (d, *J* = 8.5 Hz, 2H, arom.), 6.74 (d, *J* = 8.5 Hz, 2H, arom.), 3.72 (s, 2H, C**H**$_2$NR$_2$), 3.32 (d, *J* = 2.5 Hz, 2H, C**H**$_2$C≡CH), 2.64 (t, *J* = 2.5 Hz, 1H, CH$_2$C≡C**H**). ^{13}C NMR (75 MHz, MeOD-d_4) δ: 157.86, 131.08, 130.53, 116.19, 81.93, 73.43, 52.23, 37.22. GC-MS (EI): t_R = 5.84 min; m/z (%) 160.0 (100) $[M-H]^+$, 144.0 (13), 132.0 (45), 120.0 (40), 107.1 (95), 78.1 (46), 77.0 (58), 68.1 (14), 65.0 (17), 54.0 (29), 52.0 (30). R_f = 0.18 (cyclohexane:ethyl acetate = 7:3 (v/v) with 1% Et_3N), 0.13 (cyclohexane:ethyl acetate = 1:1 (v/v)), 0.06 (cyclohexane:ethyl acetate = 7:3 (v/v)). m.p. = 100-102 °C (dark yellow solid). HRMS (EI) cald for $C_{10}H_{11}NO$ 160.0762 $[M-H]^+$, found 160.0763.

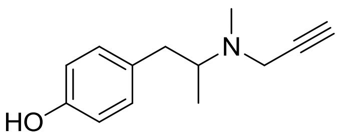

4-(2-(methyl(prop-2-yn-1-yl)amino)propyl)phenol (3)

In an oven dried, evacuated and argon purged 50 mL Schlenk

flask 601 mg 4-hydroxybenzaldehyde (4.0 mmol, 1.0 eq) were dissolved in 20 mL dry and degassed THF at RT resulting in a clear yellow solution. Subsequently, a solution of 553 mg *N*-methylpropargylamine (675 µL, 8.0 mmol, 2.0 eq) in 3 mL dry THF was added at once resulting in a dark yellow solution. The mixture was stirred at RT for 30 min. Then 1.696 g sodium triacetoxyborohydride (8.0 mmol, 2.0 eq) were added at once, followed by addition of 448 mg glacial acetic acid (458 µL, 8.0 mmol, 2.0 eq). After addition of the reducing agent the mixture became thicker and cloudy. The reaction was monitored by GC-MS and TLC analysis and carried out for 92 h at RT until nearly complete consumption of the ketone was indicated. After that time the reaction mixture was quenched with 20 mL saturated $NaHCO_3$. The solution was extracted with ethyl acetate (3 x 20 mL). The combined organic layers were washed with water (2 x 20 mL) and brine (1 x 20 mL) and dried over anhydrous Na_2SO_4. The solvent was removed in *vacuo* and the crude product purified by flash column chromatography (cyclohexane:ethyl acetate = 7:3 (v/v)) furnishing 555 mg (55%) 4-(2-(methyl(prop-2-yn-1-yl)amino)propyl)phenol (**3**) as light yellow solid. ^{1}H NMR (300 MHz, MeOD-d_4) δ: 6.99 (d, *J* = 8.4 Hz, 2H, arom.), 6.70 (d, *J* = 8.4 Hz, 2H, arom.), 3.44 (m, 2H, C**H**$_2$C≡CH), 2.95 (dd, *J* = 13.5, 2.8 Hz, 1H, C**H**$_a$H$_b$CH and m, 1H, C**H**), 2.68 (t, *J* = 2.4 Hz, 1H, CH$_2$C≡C**H**), 2.39 (s, 3H, NC**H**$_3$), 2.28 (dd, *J* = 13.7, 11.1 Hz, 1H, CH$_a$**H**$_b$CH), 0.94 (d, *J* = 6.5 Hz, 3H, C**H**$_3$). ^{13}C NMR (75 MHz, MeOD-d_4) δ: 156.75, 131.83, 131.17, 116.15, 80.29, 74.66, 60.71, 43.69, 39.08, 37.67, 14.97. GC-MS (EI): t_R = 6.39 min; m/z (%) 202.0 (0.2) $[M-H]^+$, 107.0 (19), 96.1 (100), 77.1 (16), 56.0 (30). R_f = 0.27 (cyclohexane:ethyl acetate = 1:1 (v/v)), 0.14 (cyclohexane:ethyl acetate = 7:3 (v/v). m.p. 113-115°C (light yellow solid). HRMS (EI) cald for $C_{13}H_{17}NO$ 203.1310 $[M^+]$, found 203.1314.

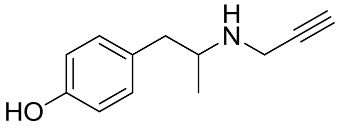

4-(2-(prop-2-yn-1-ylamino)propyl)phenol (4)

In an oven dried, evacuated and argon purged 50 mL Schlenk flask 300 mg 4-hydroxyphenylacetone (2.0 mmol, 1.0 eq) were dissolved in 5 mL dry and degassed THF at RT resulting in a clear light orange solution. Subsequently, a solution of 132 mg propargylamine (153.7 µL, 2.4 mmol, 1.2 eq) in 1 mL THF was added at once

resulting in a yellow solution. The mixture was stirred at RT for 30 min. Then 848 mg sodium triacetoxyborohydride (4.0 mmol, 2.0 eq) were added at once, followed by addition of 240 mg glacial acetic acid (229 µL, 4.0 mmol, 2.0 eq). After addition of the reducing agent the mixture became thicker and cloudy. The reaction was monitored by GC-MS and TLC analysis and carried out for 46 h at RT until nearly complete consumption of the ketone was indicated. After that time the reaction mixture was quenched with 15 mL saturated $NaHCO_3$. The solution was extracted with ethyl acetate (4 x 10 mL). The combined organic layers were washed with water (2 x 20 mL) and brine (1 x 20 mL) and dried over anhydrous Na_2SO_4. The solvent was removed in *vacuo* and the crude product purified by flash column chromatography (cyclohexane:ethyl acetate = 7:3 to 5:5 (v/v)) furnishing 293 mg (78%) 4-(2-(prop-2-yn-1-ylamino)propyl)phenol (**4**) as a yellow solid. ^{1}H NMR (300 MHz, MeOD-d_4) δ: 7.01 (d, *J* = 8.4 Hz, 2H, arom.), 6.72 (d, *J* = 8.4 Hz, 2H, arom.), 3.41 (qd, *J* = 17.0 Hz, 2.4 Hz, 2H, C**H**$_2$C≡CH), 3.08 (m, 1H, C**H**), 2.68 (dd, *J* = 13.2, 6.3 Hz, 1H, C**H**$_a$H$_b$CH), 2.58 (t, *J* = 2.4 Hz, 1H, CH$_2$C≡C**H**), 2.45 (dd, *J* = 13.2, 7.2 Hz, 1H, CH$_a$**H**$_b$CH), 1.00 (d, *J* = 6.3 Hz, 3H, C**H**$_3$). ^{13}C NMR (75 MHz, MeOD-d_4) δ: 156.99, 131.22, 130.85, 116.27, 81.88, 73.20, 54.18, 42.90, 35.84, 18.97. GC-MS (EI): t_R = 6.18 min; m/z (%) 188.1 (0.3) $[M-H]^+$, 107.0 (26), 82.1 (100), 77.1 (19), 53 (7). R_f = 0.15 (cyclohexane:ethyl acetate = 1:1 (v/v)), 0.06 (cyclohexane:ethyl acetate = 7:3 (v/v)) m.p. 107-108 °C (yellow solid). HRMS (EI) cald for $C_{12}H_{15}NO$ 189.1154 $[M^+]$, found 189.1152.

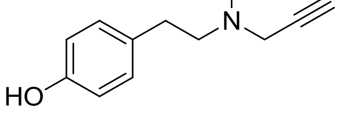

4-(2-(methyl(prop-2-yn-1-yl)amino)ethyl)phenol (5)

In an oven dried, evacuated and argon purged 100 mL Schlenk flask 691 mg (5.0 mmol, 1.0 eq) 4-hydroxyphenylethanol were dissolved at RT in 10 mL dry and degassed DMSO forming a clear light yellow solution. Subsequently, 1.265 g dry triethylamine (1.742 mL, 12.5 mmol, 2.5 eq) were added to the mixture, followed by a dropwise addition of solution of 1.989 g sulfur trioxide pyridine complex (12.5 mmol, 2.5 eq) in 10 mL dry DMSO. The resulting dark yellow mixture was let to react at RT for 1 h. Then, reaction mixture was poured onto ice-cold water (200 mL) and extracted with ethyl acetate (4

x 50 mL). The combined organic layers were washed with 10% solution of citric acid (2 x 50 mL), water (1 x 50 mL) and brine (1 x 50 mL) and dried over anhydrous sodium sulfate. The organic solvent was removed under reduced pressure resulting in a yellow oil used without further purification for the next step. Crude 4-hydroxyphenylacetaldehyde was dissolved in 15 mL of dry THF in a 50 mL Schlenk flask and 360 mg *N*-methylpropargylamine (438 µL, 6.5 mmol, 1.3 eq) were added and the mixture stirred at RT for 15 min. Then, 1.590 g sodium triacetoxyborohydride (7.5 mmol, 1.5 eq) were added at once followed by addition of 300 mg glacial acetic acid (290 µL, 5.0 mmol, 1.0 eq). After addition of the reducing agent the mixture became thicker and cloudy. The reaction was monitored by GC-MS and TLC analysis and carried out overnight for 15 h at RT until full conversion of starting material was indicated. After that time the reaction mixture was quenched with 10 mL saturated $NaHCO_3$. The solution was extracted with ethyl acatate (3 x 10 mL). The combined organic layers were washed with water (2 x 20 mL) and brine (1 x 20 mL) and dried over anhydrous Na_2SO_4. The solvent was removed *in vacuo* and the crude product purified by column chromatography (cyclohexane:ethyl acetate = 7:3 (v/v) to 5:5 (v/v)) furnishing 500 mg (53% after two steps) 4-(2-(methyl(prop-2-yn-1-yl)amino)ethyl)phenol (**5**) as a yellowish solid. ^{1}H NMR (300 MHz, MeOD-d_4) δ: 7.02 (d, *J* = 8.4 Hz, 2H, arom.), 6.70 (d, *J* = 8.4 Hz, 2H, arom.), 3.43 (d, 2H, *J* = 2.4 Hz, C**H**$_2$C≡CH), 2.69 (t, 1H, *J* = 2.4 Hz, CH$_2$C≡C**H**), 2.67 (brs, 4H, C**H**$_2$C**H**$_2$,), 2.38 (s, 3H, C**H**$_3$). ^{13}C NMR (75 MHz, MeOD-d_4) δ: 156.84, 131.54, 130.55, 116.26, 78.44, 75.38, 58.89, 45.94, 41.93, 33.66. GC-MS (EI): t_R = 6.18 min; m/z (%) 189.1 (0.3) [M^+], 107.0 (16), 91.1 (5), 82.1 (100), 77.0 (18), 53.1 (4). R_f = 0.26 (cyclohexane:ethyl acetate = 1:1 (v/v)), 0.13 (cyclohexane:ethyl acetate = 7:3 (v/v)). m.p. 116-118 °C (yellow solid). HRMS (EI) cald for $C_{12}H_{15}NO$ 189.1154 [M^+], found 189.1153.

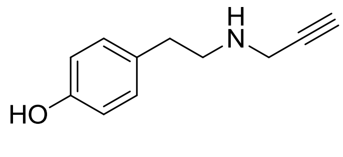

4-(2-(prop-2-yn-1-ylamino)ethyl)phenol (6)

The reaction sequence was performed in an analogous manner as described for 4-(2-(methyl(prop-2-yn-1-yl)amino)ethyl)phenol (**5**), only 358 mg propargylamine (416 µL, 6.5 mmol, 1.3 eq) was used in place of *N*-methylpropargylamine.

Flash column chromatography (cyclohexane:ethyl acetate = 8:2 (v/v) to 5:5 (v/v)) yielded 320 mg (37% after two steps) 4-(2-(prop-2-yn-1-ylamino)ethyl)phenol as a yellow solid (**6**). ^{1}H NMR (300 MHz, MeOD-d_4) δ: 7.03 (d, *J* = 8.4 Hz, 2H, arom.), 6.71 (d, *J* = 8.4 Hz, 2H, arom.), 3.38 (d, 2H, *J* = 2.4 Hz, C**H**$_2$C≡CH), 2.85 (m, 2H, CH$_2$C**H**$_2$N), 2.69 (m, 2H, C**H**$_2$CH$_2$N), 2.59 (t, *J* = 2.4 Hz, 1H, CH$_2$C≡C**H**). ^{13}C NMR (75 MHz, MeOD-d_4) δ: 156.90, 131.49, 130.58, 116.31, 81.93, 73.23, 51.07, 38.29, 35.61. GC-MS (EI): t_R = 6.135 min; m/z (%) 175.1 (3) [M^+], 107.0 (23), 91.0 (4), 77.0 (21), 68.1 (100), 53.0 (5). R_f = 0.09 (cyclohexane:ethyl acetate = 1:1 (v/v)), 0.04 (cyclohexane : ethyl acetate = 7:3 (v/v). m.p. 89-91 °C (yellow solid). HRMS (EI) cald for $C_{11}H_{13}NO$ 175.0997 [M^+], found 175.0997.

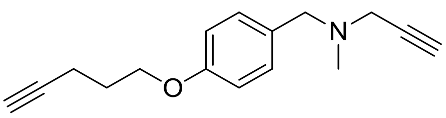

***N*-methyl-*N*-(4-(pent-4-yn-1-yloxy)benzyl)prop-2-yn-1-amine (P1)**

Method A: In an oven dried, evacuated and argon purged 10 mL Schlenk flask 87.6 mg 4-((methyl(prop-2-yn-1-yl)amino)methyl)phenol (**1**) (0.5 mmol, 1.0 eq) were dissolved in 1 mL dry and degassed THF at 0 °C (ice-water bath) resulting in a clear yellowish solution. Subsequently, 157 mg triphenylphosphine (0.6 mmol, 1.2 eq) were added, followed by dropwise addition of 121 mg diisopropyl azodicarboxylate (DIAD) (118 µL, 0.6 mmol, 1.2 eq) over the course of 5 min. Then, 50.5 mg 4-pentyn-1-ol (56 µL, 0.6 mmol, 1.2 eq) were added and the reaction mixture was stirred under inert atmosphere at RT for 22 h. After that time, the solvent was removed under reduced pressure and the crude product was purified by flash column chromatography (cyclohexane:ethyl acetate = 95:5 to 90:10 (v/v)) yielding in 102 mg (85%) *N*-methyl-*N*-(4-(pent-4-yn-1-yloxy)benzyl)prop-2-yn-1-amine (probe **P1**) as a yellowish oil.

Method B: In an oven dried, evacuated and argon purged 10 mL Schlenk flask 87.6 mg aminophenol (**1**) (0.5 mmol, 1.0 eq), 50.5 mg 4-pentyn-1-ol (46 µL, 0.6 mmol, 1.2 eq) and 157 mg triphenylphosphine (0.6 mmol, 1.2 eq) were dissolved in 0.5 mL dry and degassed THF under argon. The mixture was then sonicated for 5 min. Subsequently, 121 mg diisopropyl azodicarboxylate (DIAD) (125 µL, 0.6 mmol, 1.2 eq) were added dropwise over

the course of 5 min. The mixture was then sonicated at RT for 45 min. After that time, when no more conversion of the starting materials was observed by GC-MS and TLC analysis, the solvent was evaporated and crude product was purified by flash column chromatography (cyclohexane:ethyl acetate = 95:5 to 90:10 (v/v)) yielding 97 mg (80%) *N*-methyl-*N*-(4-(pent-4-yn-1-yloxy)benzyl)prop-2-yn-1-amine (probe **P1**) as a yellowish oil. ^{1}H NMR (300 MHz, $CDCl_3$) δ: 7.24 (d, *J* = 8.7 Hz, 2H, arom.), 6.85 (d, *J* = 8.7 Hz, 2H, arom.), 4.05 (t, *J* = 6.0 Hz, 2H, OC**H**$_2$), 3.51 (s, 2H, Ph-C**H**$_2$N), 3.29 (d, *J* = 2.4 Hz, 2H, - NC**H**$_2$C≡CH), 2.40 (td, *J* = 7.0 Hz, 2.7 Hz, 2H, C**H**$_2$C≡CH), 2.33 (s, 3H, C**H**$_3$), 2.27 (t, *J* = 2.4 Hz, 1H, NCH$_2$C≡C**H**), 1.99 (quint, *J* = 6.6 Hz, 2H, CH$_2$C**H**$_2$CH$_2$), 1.96 (t, *J* = 2.7 Hz, 1H, CH$_2$C≡C**H**). ^{13}C NMR (75 MHz, $CDCl_3$) δ: 158.31, 130.55, 130.50, 114.47, 83.66, 78.72, 73.45, 68.96, 66.29, 59.41, 44.73, 41.77, 28.37, 15.33. GC-MS (EI): t_R = 6.75 min; m/z (%) 241.2 (23) [M$^+$], 198.1 (14), 173.1 (94), 158.1 (3), 134.0 (8), 107.0 (100), 82.1 (12), 77.0 (9), 65.1 (5), 51.1 (2). R_f = 0.36 (cyclohexane:ethyl acetate = 7:3 (v/v), 0.13 (cyclohexane:ethyl acetate = 9:1 (v/v)). HRMS (EI) cald for $C_{16}H_{19}NO$ 241.1467 [M$^+$], found 241.1463.

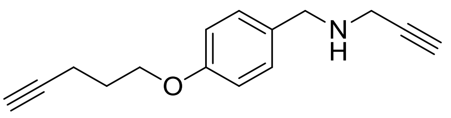

_N_-(4-(pent-4-yn-1-yloxy)benzyl)prop-2-yn-1-amine (P2)

In an oven dried, evacuated and argon purged 10 mL Schlenk flask 162 mg 4-((prop-2-yn-1-ylamino)methyl)phenol (**2**) (1.0 mmol, 1.0 eq) and 101 mg 4-pentyn-1-ol (112 µL, 1.2 mmol, 1.2 eq) were dissolved in 2 mL dry and degassed THF at RT. Subsequently, 315 mg triphenylphosphine (1.2 mmol, 1.2 eq) were added and the mixture cooled to 0 °C in an ice-water bath. Then 243 mg diisopropyl azodicarboxylate (DIAD) (236 µL, 1.2 mmol, 1.2 eq) were added dropwise over the course of 5 min and the reaction mixture was stirred under argon at RT for 25 h. After that time the solvent was removed under reduced pressure and the crude product was purified by flash column chromatography (cyclohexane:ethyl acetate = 3:2 (v/v)) yielding 190 mg (84%) *N*-(4-(pent-4-yn-1-yloxy)benzyl)prop-2-yn-1-amine (probe **P2**) as a yellow oil. ^{1}H NMR (300 MHz, MeOD-d$_4$) δ: 7.24 (d, *J* = 8.7 Hz, 2H, arom.), 6.88 (d, *J* = 8.7 Hz, 2H, arom.), 4.06 (t, *J* = 6.1 Hz, 2H, OC**H**$_2$), 3.76 (s, 2H, Ph-C**H**$_2$N), 3.33 (d, *J* =

2.4 Hz, 2H, NC**H**$_2$C≡CH), 2.64 (t, *J* = 2.4 Hz, 1H, NCH$_2$C≡C**H**), 2.40 (td, *J* = 7.0 Hz, 2.4 Hz, 2H, CH$_2$C**H**$_2$C≡CH), 2.24 (t, *J* = 2.4 Hz, 1H, CH$_2$C≡C**H**), 1.95 (quint, *J* = 6.6 Hz, 2H, CH$_2$C**H**$_2$CH$_2$). ^{13}C NMR (125 MHz, MeOD-d$_4$) δ: 159.76, 132.00, 131.03, 115.51, 84.13, 81.97, 73.41, 69.98, 67.32, 52.17, 37.29, 29.51, 15.74. GC-MS (EI): t_R = 6.79 min; m/z (%) 226.1 (95) [M-H]$^+$, 198.1 (87), 173.1 (30), 160.1 (38), 144.1 (25), 131.1 (12), 120.1 (19), 107.0 (100), 89.1 (9), 77.1 (19), 68.1 (13), 51.1 (5). R$_f$ = 0.29 (cyclohexane:ethyl acetate = 1:1 (v/v), 0.20 (cyclohexane:ethyl acetate = 3:2 (v/v)). HRMS (EI) cald for $C_{15}H_{17}NO$ 226.1232 [M-H]$^+$, found 226.1228.

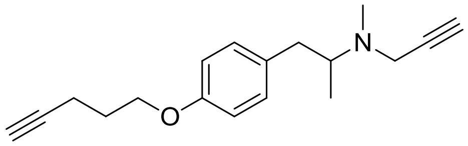

_N_-methyl-_N_-(1-(4-(pent-4-yn-1-yloxy)phenyl)propan-2-yl)prop-2-yn-1-amine (P3)

In an oven dried, evacuated and argon purged 10 mL Schlenk flask 203 mg 4-(2-(methyl(prop-2-yn-1-yl)amino)propyl)phenol (**3**) (1.0 mmol, 1.0 eq) were dissolved in 2 mL dry and degassed THF at 0 °C (ice-water bath) resulting in a clear yellow solution. Subsequently, 315 mg triphenylphosphine (1.2 mmol, 1.2 eq) were added, followed by dropwise addition of 243 mg diisopropyl azodicarboxylate (DIAD) (236 µL, 1.2 mmol, 1.2 eq) over the course of 5 min. Then, 101 mg 4-pentyn-1-ol (112 µL, 1.2 mmol, 1.2 eq) were added and the reaction mixture was stirred under inert atmosphere at RT for 16 h. After that time, the solvent was removed *in vacuo* and the crude product was purified by flash column chromatography (cyclohexane:ethyl acetate = 9:1 to 3:2 (v/v)) furnishing 181 mg (67%) *N*-methyl-*N*-(1-(4-(pent-4-yn-1-yloxy)phenyl)propan-2-yl)prop-2-yn-1-amine (probe **P3**) as a yellow oil. ^{1}H NMR (500 MHz, MeOD-d$_4$) δ: 7.08 (d, *J* = 8.5 Hz, 2H, arom.), 6.84 (d, *J* = 8.5 Hz, 2H, arom.), 4.04 (t, *J* = 6.0 Hz, 2H, OC**H**$_2$), 3.44 (m, 2H, C**H**$_2$C≡CH), 3.02 – 2.91 (m, 2H, C**H**$_a$H$_b$CH, CH$_a$H$_b$C**H**), 2.67 (t, *J* = 2.4 Hz, 1H, CH$_2$C≡C**H**), 2.40 (s, 3H, NC**H**$_3$), 2.37 (td, *J* = 7.1 Hz, 2.6 Hz, 2H, CH$_2$C**H**$_2$C≡CH) 2.32 (dd, *J* = 11.5 Hz, 8.8 Hz,1H, CH$_a$**H**$_b$CH), 2.24 (t, *J* = 2.7 Hz, 1H), 1.94 (quint, *J* = 6.6 Hz, 2H, CH$_2$C**H**$_2$CH$_2$), 0.94 (d, *J* = 6.6 Hz, 3H, C**H**$_3$). ^{13}C NMR (125 MHz, MeOD-d$_4$) δ: 158.84, 133.22, 131.19, 115.50, 84.17, 80.33, 74.64, 69.96, 67.32, 60.63, 43.67, 39.08, 37.66, 29.55, 15.76, 15.02. GC-MS (EI): t_R = 7.24 min; m/z (%)

268.2 (0.2) $[M\text{-}H]^+$, 229.1 (1), 201.1 (1), 173.1 (1), 144.1 (1), 134.1 (1), 107.0 (7), 96.1 (100), 77.1 (2), 56.1 (14). R_f = 0.27 (cyclohexane:ethyl acetate = 7:3 (v/v)), 0.07 (cyclohexane:ethyl acetate = 9:1 (v/v)). HRMS (EI) cald for $C_{18}H_{23}NO$ 269.1780 $[M^+]$, found 269.1774.

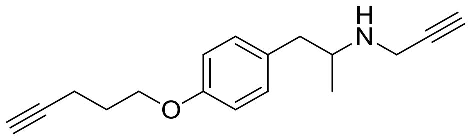

***N*-(1-(4-(pent-4-yn-1-yloxy)phenyl)propan-2-yl)prop-2-yn-1-amine (P4)**

In an oven dried, evacuated and argon purged 10 mL Schlenk flask 189 mg 4-(2-(prop-2-yn-1-ylamino)propyl)phenol (4) (1.0 mmol, 1.0 eq) were dissolved in 2 mL dry and degassed THF at 0 °C (ice-water bath) resulting in a clear yellow solution. Subsequently, 315 mg triphenylphosphine (1.2 mmol, 1.2 eq) were added, followed by a dropwise addition of 243 mg diisopropyl azodicarboxylate (DIAD) (236 µL, 1.2 mmol, 1.2 eq) over the course of 5 min. Then, 101 mg 4-pentyn-1-ol (112 µL, 1.2 mmol, 1.2 eq) were added and the reaction mixture was stirred under inert atmosphere at RT for 24 h. After that time, the solvent was removed *in vacuo* and the crude product was purified by flash column chromatography (cyclohexane:ethyl acetate = 7:3 (v/v)) furnishing 117 mg (46%) *N*-(1-(4-(pent-4-yn-1-yloxy)phenyl)propan-2-yl)prop-2-yn-1-amine (probe **P4**) as a yellow oil. ^{1}H NMR (300 MHz, MeOD-d_4) δ: 7.10 (d, *J* = 8.4 Hz, 2H, arom.), 6.86 (d, *J* = 8.4 Hz, 2H, arom.), 4.04 (t, *J* = 6.0 Hz, 2H, OC**H**$_2$), 3.42 (qd, *J* = 17.1 Hz, 1.8 Hz, 2H, NC**H**$_2$C≡CH), 3.16 – 3.03 (m, 1H, C**H**), 2.72 (dd, *J* = 13.2, 6.3 Hz, 1H, C**H**$_a$H$_b$CH), 2.59 (t, *J* = 2.4 Hz, 1H, NCH$_2$C≡C**H**), 2.48 (dd, *J* = 13.4, 7.6 Hz, 1H, CH$_a$**H**$_b$CH), 2.37 (td, *J* = 7.0 Hz, 2.4 Hz, 2H, CH$_2$C**H**$_2$C≡CH), 2.24 (t, *J* = 2.4 Hz, 1H, CH$_2$CH$_2$C≡C**H**), 1.95 (quint, *J* = 6.6 Hz, 2H, CH$_2$C**H**$_2$CH$_2$), 1.00 (d, *J* = 6.3 Hz, 3H, NC**H**$_3$). ^{13}C NMR (75 MHz, MeOD-d_4) δ: 159.05, 132.24, 131.25, 115.58, 84.16, 81.91, 73.20, 69.96, 67.32, 54.12, 42.86, 35.84, 29.55, 18.99, 15.76. GC-MS (EI): t_R = 7.05 min; m/z (%) 254.2 (0.2) $[M\text{-}H]^+$, 201.1 (1), 174.1 (6), 145.1 (1), 133.1 (1), 107.1 (7), 82.1 (100), 77.1 (4), 65.1 (2), 51.1 (1). R_f = 0.25 (cyclohexane : ethyl acetate = 3:2 (v/v), 0.17 (cyclohexane:ethyl acetate = 7:3 (v/v)). HRMS (EI) cald for $C_{17}H_{21}NO$ 255.1623 $[M^+]$, found 255.1626.

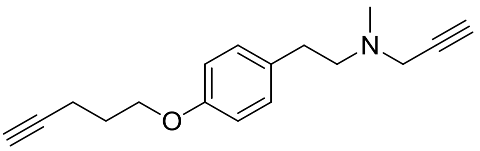

N-methyl-N-(4-(pent-4-yn-1-yloxy)phenethyl)prop-2-yn-1-amine (P5)

In an oven dried, evacuated and argon purged 10 mL Schlenk flask 189 mg 4-(2-(methyl(prop-2-yn-1-yl)amino)ethyl)phenol (**5**) (1.0 mmol, 1.0 eq) and 101 mg 4-pentyn-1-ol (112 µL, 1.2 mmol, 1.2 eq) were dissolved in 2 mL dry and degassed THF at RT. Subsequently, 315 mg triphenylphosphine (1.2 mmol, 1.2 eq) were added and the mixture cooled to 0 °C in an ice-water bath. Then 243 mg diisopropyl azodicarboxylate (DIAD) (236 µL, 1.2 mmol, 1.2 eq) were added dropwise over the course of 5 min and the reaction mixture was stirred under argon at RT for 26 h. After that time the solvent was removed under reduced pressure and the crude product was purified by flash column chromatography (cyclohexane:ethyl acetate = 3:2 (v/v)) yielding 178 mg (70%) *N*-methyl-*N*-(4-(pent-4-yn-1-yloxy)phenethyl)prop-2-yn-1-amine (probe **P5**) as a yellowish oil. ^{1}H NMR (300 MHz, MeOD-d$_4$) δ: 7.13 (d, *J* = 8.7 Hz, 2H, arom.), 6.86 (d, *J* = 8.7 Hz, 2H, arom.), 4.05 (t, *J* = 6.0 Hz, 2H, OC**H**$_2$), 3.42 (d, 2H, *J* = 2.4 Hz, C**H**$_2$C≡CH), 2.70 (m, 5H, C**H**$_2$C**H**$_2$, CH$_2$C≡C**H**), 2.45-2.33 (m, 5H: 2.38 (s, 3H, C**H**$_3$), 2.37 (td, *J* = 7.0 Hz, 2.7 Hz, 2H, CH$_2$C**H**$_2$C≡CH)), 2.26 (t, *J* = 2.7 Hz, 1H, CH$_2$CH$_2$C≡C**H**), 1.96 (quint, *J* = 6.6 Hz, 2H, CH$_2$C**H**$_2$CH$_2$). ^{13}C NMR (75 MHz, MeOD-d$_4$) δ: 158.90, 133.07, 130.57, 115.61, 84.16, 78.65, 75.17, 69.95, 67.33, 58.80, 45.95, 41.99, 33.76, 29.54, 15.75. GC-MS (EI): t_R = 7.06 min; m/z (%) 255.2 (0.4) [M$^+$], 187.1 (1), 173.1 (1), 121.1 (2), 107.1 (6), 91.1 (2), 82.1 (100), 77.1 (3), 65.1 (2), 51.1 (1). R_f = 0.27 (cyclohexane:ethyl acetate = 3:2 (v/v)), 0.22 (cyclohexane:ethyl acetate = 7:3 (v/v)). HRMS (EI) cald for $C_{17}H_{21}NO$ 255.1623 [M$^+$], found 255.1625.

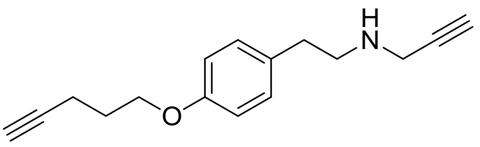

***N*-(4-(pent-4-yn-1-yloxy)phenethyl)prop-2-yn-1-amine (P6)**

In an oven dried, evacuated and argon purged 10 mL Schlenk flask 175 mg 4-(2-(prop-2-yn-1-ylamino)ethyl)phenol (**6**) (1.0 mmol, 1.0 eq) and 101 mg 4-pentyn-1-ol (112 µL, 1.2 mmol, 1.2 eq) were dissolved in 2 mL dry and degassed THF at RT. Subsequently, 315 mg

triphenylphosphine (1.2 mmol, 1.2 eq) were added and the mixture cooled down to 0 °C on ice-water bath. Then 243 mg diisopropyl azodicarboxylate (DIAD) (236 µL, 1.2 mmol, 1.2 eq) were added dropwise over the course of 5 min and the reaction mixture was stirred under argon at RT for 26 h. After that time the solvent was removed under reduced pressure and the crude product was purified by flash column chromatography (cyclohexane:ethyl acetate = 3:2 to 1:1 (v/v)) yielding 220 mg (91%) *N*-(4-(pent-4-yn-1-yloxy)phenethyl)prop-2-yn-1-amine (probe **P6**) as a yellowish oil. ^{1}H NMR (300 MHz, MeOD-d_4) δ: 7.12 (d, *J* = 8.7 Hz, 2H, arom.), 6.85 (d, *J* = 8.7 Hz, 2H, arom.), 4.03 (t, *J* = 6.0 Hz, 2H, OC**H**$_2$), 3.38 (d, 2H, *J* = 2.1 Hz, C**H**$_2$C≡CH), 2.87 (m, 2H, C**H**$_2$NH), 2.72 (m, 2H, C**H**$_2$CH$_2$NH), 2.60 (t, 1H, *J* = 2.4 Hz, CH$_2$C≡C**H**), 2.36 (td, *J* = 7.1, 2.7 Hz, 2H, CH$_2$C**H**$_2$C≡CH), 2.24 (t, *J* = 2.7 Hz, 1H, CH$_2$CH$_2$C≡C**H**), 1.94 (quint, *J* = 6.6 Hz, 2H, CH$_2$C**H**$_2$CH$_2$). ^{13}C NMR (75 MHz, MeOD-d_4) δ: 158.94, 132.88, 130.60, 115.66, 84.17, 81.95, 73.24, 69.97, 67.32, 50.98, 38.30, 35.61, 29.52, 15.75. GC-MS (EI): t_R = 7.03 min; m/z (%) 241.2 (0.2) [M$^+$], 187.1 (1), 174.1 (56), 159.1 (1), 146.1 (4), 134.1 (1), 107.0 (20), 91.1 (4), 77.1 (7), 68.1 (100), 51.1 (2). R_f = 0.21 (cyclohexane:ethyl acetate = 1:1 (v/v)), 0.13 (cyclohexane:ethyl acetate = 3:2 (v/v)). HRMS (EI) cald for $C_{16}H_{19}NO$ 241.1467 [M$^+$], found 241.1471.

Biochemical experiments

Materials for Biological Experiments

Cell line DBTRG-05MG (ATCC code CRL2020) was obtained from American Type Culture Collection (ATCC, Manassas, VA). Cell line RAEW was generously donated by Dr. Walter Berger (Institute of Cancer Research, Medical University of Vienna, Austria). All cell culture media and supplements were obtained from PAA, Gibco or Sigma-Aldrich. Human recombinant MAO A and MAO B were generously provided by Prof. D. E. Edmondson (Department of Biochemistry and Chemistry, EmoryUniversity, Atlanta, USA). Anti-MAO A (ab90675) and anti-MAO B (ab67297) antibodies were purchased from Abcam. Amplex® Red MAO assay kit (A12214) was purchased from Invitrogen, kynuramine·2HBr,

benzylamine·HCl, deprenyl·HCl, pargyline·HCl, avidin-agarose beads and Thiazolyl Blue Tetrazolium Bromide were obtained from Sigma-Aldrich.

Cell culture

The human glioblastoma multiforme (RAEW (established from a surgical specimen and analysed in early passage) or DBTRG-05MG) cells were cultured in RPMI 1640 medium containing 2 mM L-glutamine, 100 µM penicillin/streptomycin supplemented with 10% FCS in a humidified 5% CO_2 incubator at 37 °C.

***In situ* ABPP labeling experiments**

Method A: For analytical and preparative *in situ* labeling experiments cells were grown to ca. 80-90% confluency in a complete medium on Petri dishes (150 x 25 mm). Then the medium was aspirated and 20 mL fresh medium containing either DMSO (as blank control) or probes **P1** or **P3** in the appropriate concentration (10-200 µM) were added. DMSO content in the medium did not exceed 0.02%. Cells were incubated for 2 h with varying concentrations of probes **P1** and **P3** at 37 °C and 5% CO_2 for analytical studies and with concentration of 100 µM for preparative studies. Subsequently, the medium was carefully aspirated, cells were washed with 10 mL PBS to remove the excess of the probe and then harvested in 20 mL fresh PBS by scraping. Cell pellets were isolated by centrifugation (1200 rpm, 5 min, 4 °C), resuspended in 500 µL PBS and lysed by sonication under ice cooling. Soluble and insoluble fraction were separated by centrifugation at 14 800 rpm for 60 min at 4 °C. Insoluble pellets were resuspended in 500 µL PBS by sonication under ice cooling. Protein concentration was assayed (DC Protein Bioassay Kit, Bio-Rad) and adjusted to 2 mg/mL in PBS.

Method B: Cells were grown to ca. 80-90% confluency in a complete medium on Petri dishes (150 x 25 mm). Then the medium was aspirated and cells were washed with 10 mL PBS and then harvested in 20 mL fresh PBS by scraping. Cell pellets were isolated by centrifugation (800 g, 5 min), resuspended in 500 µL complete medium containing probes **P1** or **P3** at the appropriate concentration (10 – 200 µM, from 50 mM DMSO stock) and cells were incubated for 2 h at 37 °C at 900 rpm. Subsequently, cell pellets were spun for 5 min at 800 g at RT to

remove the medium with excess of the probe, washed twice with 500 µL PBS, resuspended in 500 µL PBS and processed further as described for Method A. Method B allowed using smaller volumes of media as well as of DMSO stocks of probes giving results identical to Method A.

In situ **competitive ABPP experiments**

Cells were grown to ca. 80-90% confluency in a complete medium on Petri dishes (150 x 25 mm). Then the medium was aspirated and cells were washed with 10 mL PBS and then harvested in 20 mL fresh PBS by scraping. Cell pellets were isolated by centrifugation (800 g, 5 min), resuspended in 500 µL complete medium containing 10 mM pargyline hydrochloride or deprenyl hydrochloride (5 µL, 1 M stock in water, 100-fold excess) and incubated for 1 h at 37 °C at 900 rpm. Then, 100 µM probe **P1** or **P3** (1 µL, 50 mM stock in DMSO) was added to the medium and cells were incubated for 1 h at 37 °C at 900 rpm. Subsequently, cell pellets were spun for 5 min at 800 g at RT to remove the medium with excess of the inhibitor and the probe, washed twice with 500 µL PBS, resuspended in 500 µL PBS and lysed by sonication under ice cooling. Soluble and insoluble fraction were separated by centrifugation at 14 800 rpm for 60 min at 4 °C. Insoluble pellets were resuspended in 500 µL PBS by sonication under ice cooling. Protein concentration was assayed (DC Protein Bioassay Kit, Bio-Rad) and adjusted to 2 mg/mL in PBS.

Cycloaddition Reactions, Protein Electrophoresis and In-Gel Fluorescence Scanning

In analytical experiments 44 µL probe-bound cell lysate (2 mg/mL protein concentration) was used to append a fluorescent reporter tag *via* click chemistry (CC) reaction.[1-4] 100 µM (5,6)-TAMRA-azide (Figure S2 A) (1 µL / reaction, 5 mM stock in DMSO) was added to each lysate sample followed by 1 mM TCEP (1 µL / reaction, 50 mM stock in water) and 100 µM TBTA ligand (3 µL / reaction, 1.7 mM stock in DMSO:*tert*-BuOH 1:4 (v/v)). Samples were gently vortexed and 1 mM $CuSO_4$ (1 µL / reaction, 50 mM stock in water) was added to initiate the 1,3-cycloaddition reaction, giving the total reaction volume of 50 µL. Samples were incubated at RT for 1 h at 350 rpm in the dark. Then, the reaction was quenched with 50 µL 2 x SDS

loading buffer and samples denaturated for 5 min at 95 °C. Proteins (50 µL sample) were separated by SDS-PAGE on 10% polyacrylamide gels and fluorescence was recorded in a Fujifilm Las-4000 Luminescent Image Analyzer with a Fujinon VRF43LMD3 Lens and a 575DF20 filter. Gels were then subjected to Coomassie Brilliant Blue staining to verify equivalent protein loading.

Reactions for preparative enrichment were carried out together with a control lacking the probe to subtract the background unspecific protein binding on avidin-agarose beads from the specifically biotin-avidin enriched protein samples. For preparative scale click chemistry reactions 955 µL probe-bound cell lysates were mixed with 50 µM biotin-TAMRA-azide[5] (Figure S2 B) (5 µL, 10 mM stock in DMSO), 500 µM TCEP (10 µL, 50 mM stock in water), 50 µM TBTA ligand (30 µL, 1.7 mM stock in DMSO:*tert*-BuOH 1:4 (v/v)) and 500 µM $CuSO_4$ (10 µL, 50 mM stock in water). Samples were gently vortexed and incubated for 1 h at RT in the dark under constant mixing. After CC, proteins were precipitated using three volumes of pre-chilled acetone. Samples were stored at -21 °C overnight and centrifuged at 14 800 rpm for 20 min at 4 °C. The supernatant was discarded, and the pellet was washed twice with pre-chilled methanol (200 µL) and resuspended by sonication (Bandelin Sonopuls, 5 sec, 10% intensity) under ice cooling. Subsequently, the pellet was warmed up to RT and dissolved in 0.4% SDS in PBS (800 µL) by sonication (Bandelin Sonopuls, 6 x 10 sec, 30% intensity) and incubated under gentle mixing with 60 µL of pre-equilibrated avidin-agarose beads for 1 h at RT. Thereafter, the beads were washed with 0.2% SDS in PBS (3 x 1 mL), with urea (2 x 1 mL, 6 M) and with PBS (3 x 1 mL). After each washing, the beads were spun for 1 min at 2 000 rpm and supernatant carefully discarded. 60 µL of 2×SDS loading buffer was added to the beads and the proteins were released from the beads for the preparative SDS-PAGE by incubation for 6 min at 90 °C. The beads were spun down for 2 min at 13 000 rpm and supernatant containing released proteins were subjected to SDS-PAGE and in-gel fluorescence scanning. Gel bands with proteins of interest were excised, washed, and tryptically digested as described previously.[6]

Mass spectrometry and bioinformatics

ESI-MS spectra were recorded by a Thermo LTQ Orbitrap XL coupled to a Dionex UltiMate 3000 RSLC nano. The peptides were loaded on a Dionex Acclaim® PepMap 100 75 µm × 2 cm, C18 (3 µm) and subsequently eluted and separated by a Dionex Acclaim® PepMap RLSC 75 µm × 15 cm, C18 (2 µm).

Mass spectrometry data was searched against the corresponding databases via the software Proteome Discoverer 1.3 (Thermo Scientific) using the SEQUEST algorithm.[7] The search was limited to only tryptic peptides, two missed cleavage sites, precursor mass tolerance of 10 ppm and fragment mass tolerance of 0.8 Da. Filters were set to further refine the search results. The X_{corr} vs. charge state filter was set to X_{corr} values of 1.5, 2.0, 2.25 and 2.5 for charge states +1, +2, +3 and +4, respectively. The number of different peptides had to be ≥ 2 and the peptide confidence filter was set to at least medium. These filter values are similar to others previously reported for SEQUEST analysis. X_{corr} values (Score) of each run, the peptide spectrum matches (PSM) as well as the total number of obtained peptides and unique peptides are reported in Supporting Table 1.

Western Blotting

For MAO B detection, the cell lysate samples were prepared in the identical way as for in-gel fluorescence scanning. The proteins separated by 10% SDS-PAGE were transferred to a nitrocellulose membrane (VWR) with a semi-dry blotter for 1 h at 140 mA. The blots were saturated with 5% non-fat dried milk in TBST (TBS with 0.05% Tween 20®, pH 8.0) for 1 h at 4 °C, incubated with primary rabbit polyclonal anti-MAO B antibody (1:20 000 dilution in 1% milk in TBST) overnight at 4 °C, washed five times 5 min with TBST and detected with goat anti-rabbit IgG HRP-conjugated secondary antibody (1:20 000 dilution in 1% milk in TBST, 45 min at RT). Signals were detected using Amersham ECL Plus Western Blotting Detection reagents (GE Healthcare) or SuperSignal® West Pico Chemiluminescent Substrate (Thermo Scientific) and exposure to light-sensitive film (Kodak or Agfa).

Labeling of human recombinant monoamine oxidase (MAO A and B) *in vitro*

Pichia pastoris membrane preparations overexpressing human monoamine oxidase MAO A and B were diluted with 50 mM potassium phosphate buffer, pH 7.5 up to the concentration of ca. 1 mg/mL. Protein samples (43 µL) were incubated with 10 µM probe (**P1**-**P6**) (1 µL of 500 µM stock in DMSO) for 1 h at RT with gentle mixing at 750 rpm. For heat controls, protein samples were denaturated at 96 °C for 6 min and then cooled down to RT prior to the addition of a probe. The samples (44 µL) were then subjected to CC and SDS-PAGE analysis as described previously. In competitive ABPP labeling experiments 42 µL protein sample were incubated for 30 min at RT with 1 µL given MAO inhibitor (10-100 fold probe excess, 0.1 – 1.0 mM, 5 – 50 mM stock in water), followed by addition of 1 µL 10 µM probe which was incubated as described above.

MAO Activity Measurements

Activity of MAO A and MAO B was assigned in the continuous Amplex® Red – HRP coupled assay using kynuramine and benzylamine as substrates for MAO A and MAO B, respectively. The activity was assayed spectrophotometrically by monitoring the rate of resorufin formation at 560 nm (product of Amplex Red oxidation). To 3.5 µg MAO A or 2.5 µg MAO B in 50 µL 50 mM potassium phosphate buffer (pH 7.5) were added 50 µL chromogenic solution containing 3 µL 100 mM stock of kynuramine dihydrobromide (for MAO A) or benzylamine hydrochloride (for MAO B), 1 µL 100 U/mL stock horse radish peroxidase, 1 µL 5 mM stock Amplex® Red (10-acetyl-3,7-dihydroxyphenoxazine) and 45 µL 50 mM potassium phosphate buffer. The samples were mixed thoroughly and absorbance change was recorded in a 96-well Flat Bottom Nunclon Surface (Brand) plate at 560 nm at 25 °C by a TECAN Infinite M200pro. All measurements were performed in triplicates.

IC_{50} Measurements

IC_{50} values for the inhibition of MAO A and MAO B by probes **P1**, **P3** as well as by pargyline and deprenyl and were measured by the Amplex® Red – peroxidase coupled assay described above. 3.5 µg MAO A or 2.5 µg MAO B were incubated in 50 µL 50 mM potassium

phosphate buffer (pH 7.5) with varying concentrations of probes **P1**, **P3**, MAO inhibitors or DMSO as control for 1 h at 25 °C under gentle shaking (750 rpm). Then 50 µL chromogenic solution described above were added to give a total volume of 100 µL, the solution well mixed and absorbance change was recorded in a 96-well Flat Bottom Nunclon Surface (Brand) plate at 560 nm at 25 °C by a TECAN Infinite M200pro. All measurements were performed in triplicates. IC_{50} values were calculated from curve fittings (Boltzmann model) by Origin Pro 8.6 (OriginLab Corporation).

Cytotoxicity determination / MTT assay

DBTRG-05MG cell line was cultured in RPMI 1640 medium (2 mM L-glutamine) supplemented with 10% FBS. Cells from subconfluent cultures were used for the assay. Precisely, 7.5 x 10^3 CRL2020 cells were plated in 96 well flat-bottom plates (Nunclon, Nunc) in 100 µL medium and cultured for 24 h to obtain 30-40% confluent cultures. Probes **P1** and **P3** were diluted 1:100 from 100 x DMSO stocks in 100 µL of the appropriate culture medium and added to the cells after careful removal of the blank culture medium. After 24 h incubation 20 µL MTT substrate solution (5mg/mL in PBS) were added. Following 2 h incubation, the medium was discarded and cells were lysed in 200 µL DMSO. The complete dissolution of the formazan salt (metabolic product of MTT) was checked under the microscope and the optical density was measured at 570 nm (background subtraction at 630 nm) by a TECAN Infinite M200pro. All measurements were performed in triplicates. EC_{50} values were calculated from curve fittings by Origin Pro 8.6 (OriginLab Corporation).

Supporting figures

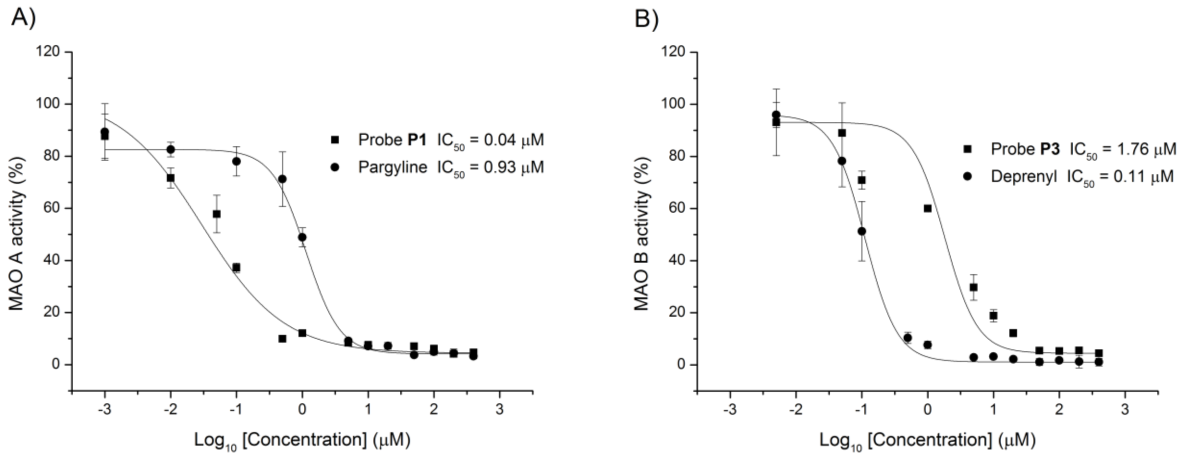

Figure S1. Inhibition of MAO A by probe **P1** and pargyline (A) and MAO B by probe **P3** and deprenyl (B). IC_{50} values were determined using curve fittings (Boltzmann model) by Origin Pro 8.6 (OriginLab Corporation).

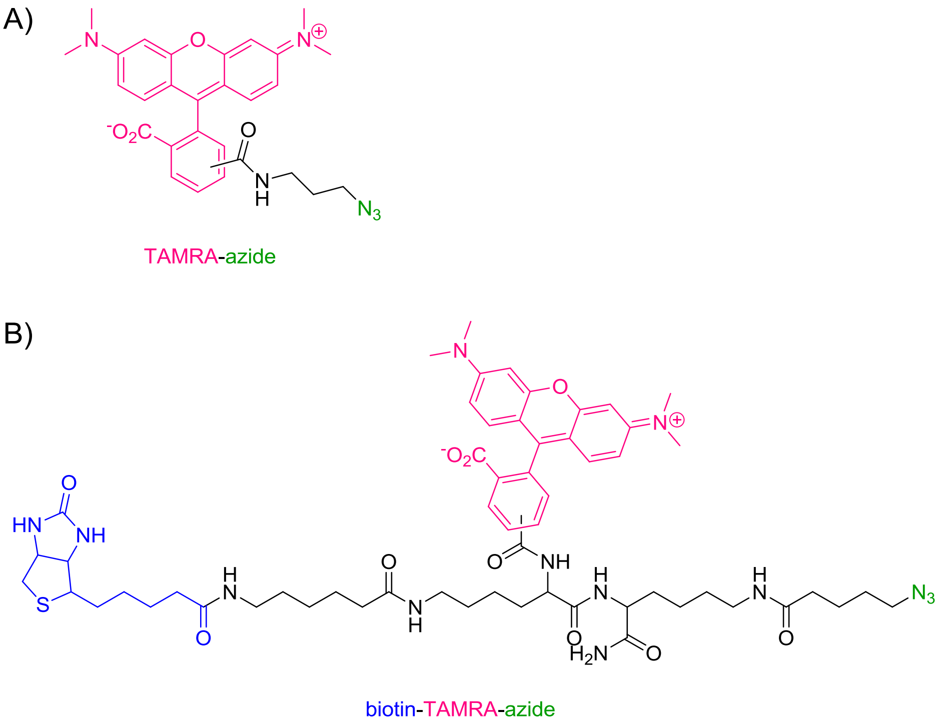

Figure S2. Structure of TAMRA-N_3 fluorescent tag (A) used for visualization and biotin-TAMRA-N_3 trifunctional tag (B) used for enrichment and visualization of labeled enzyme targets.

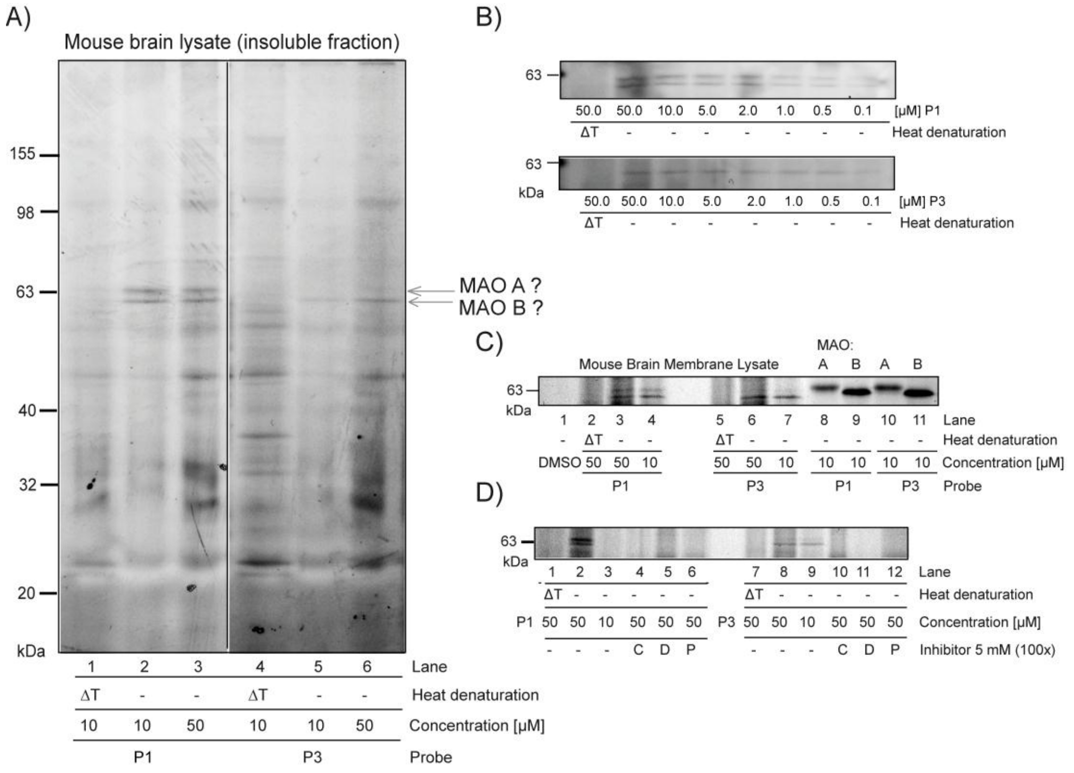

Figure S3. A) Labeling of mouse brain homogenate (insoluble fraction) with probes **P1** and **P3**. B) Concentration-dependent labeling of mouse brain membrane lysate by probe **P1** and **P3**. C) Comparative labeling of mouse brain lysate (membrane fraction) and MAO A and MAO B with probes **P1** and **P3**. D) Competitive labeling of mouse brain lysate with probes **P1** and **P3** and MAO specific irreversible inhibitors (C – clorgyline, D – deprenyl, P – pargyline).

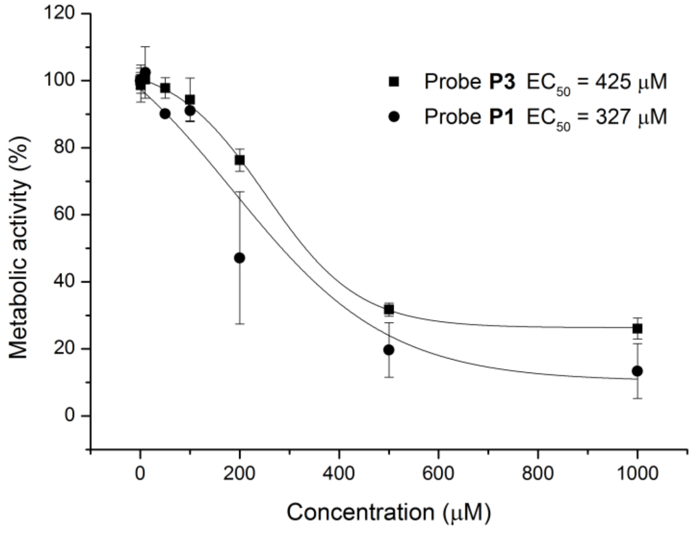

Figure S4. Cell toxicity assigned by MTT assay for metabolic activity on human brain tumor cell line DBTRG-05MG.

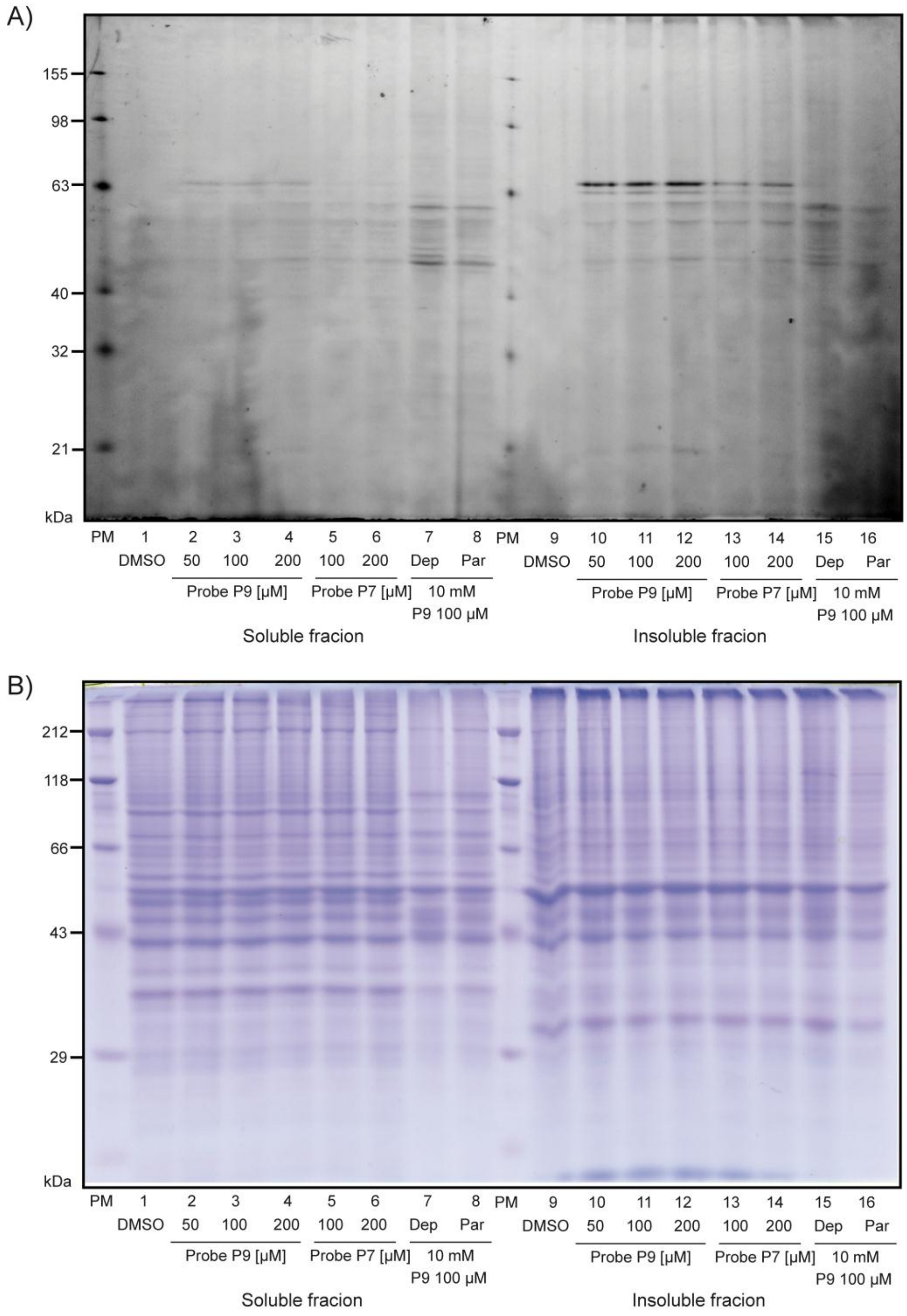

Figure S5. Fluorescent SDS-PAGE analysis of *in situ* labeling of human brain cancer cell line RAEW with probes **P1** and **P3**. A) *In situ* ABPP labeling of human brain cancer cells with probes **P1** and **P3** and competitive labeling with MAO inhibitors deprenyl (Dep) and pargyline (Par) and probe **P3.** (B) Coomassie Brilliant Blue (CBB) staining after in-gel fluorescence scanning.

Supporting Table 1. Protein identified by mass spectrometry

Protein	Protein ID	MW	Score	Peptides	Unique Peptides	PSM
Amine oxidase [flavin-containing] A	IPI00008483.1	59.6435	158.79	17	17	52

List of abbreviations

ABPP	Activity-Based Protein Profiling
ATCC	American Type Culture Collection
CAM	cerium ammonium molybdate
CC	click chemistry
DCE	1,2-dichloroethane
DMSO	dimethyl sufoxide
DI	direct inlet
EI	electron impact
ESI	electrospray ionization
FAD	flavin adenine dinucleotide
FCS	fetal calf serum
GC	gas chromatography
HRP	horse radish peroxidase
HRMS	high resolution mass spectrometry
LC	liquid chromatography
MAO	monoamine oxidase
MS	mass spectrometry
MTT	Thiazolyl Blue Tetrazoliumbromide
PBS	phosphate buffered saline
PSM	peptide spectrum match
SDS-PAGE	sodium dodecyl polyacrylamide electrophoresis
TAMRA	5(6)-carboxytetramethylrhodamine
TBTA	tris[(1-benzyl-1*H*-1,2,3-triazol-4-yl)methyl] amine
TBS	tris buffered saline
TCEP	tris(2-carboxyethyl)phosphine
TLC	thin layer chromatography
TOF	time-of-flight
THF	tetrahydrofuran

References

[1] T. Boettcher, S. A. Sieber, *J. Am. Chem. Soc.* **2010**, *132*, 6964-6972.

[2] T. Boettcher, S. A. Sieber, *Angew. Chem.* **2008**, 120, 4677-4680; *Angew. Chem. Int. Ed.* **2008**, *47*, 4600-4603.

[3] A. E. Speers, B. F. Cravatt, *Chem. Biol.* **2004**, *11*, 535-546.

[4] A. E. Speers, G. C. Adam, B. F. Cravatt,*J. Am. Chem. Soc.***2003**, *125*, 4686-4687.

[5] J. Eirich, J. L. Burkhart, A. Ullrich, A. Vollmar, S. Zahler, U. Kazmaier, S. A. Sieber, submitted, **2012**

[6] S. A. Sieber, S. Niessen, H. S. Hoover, B. F. Cravatt, *Nat. Chem. Biol.* **2006**, *2*, 274-281.

[7] S. P. Mirza, B. D. Halligan, A. S. Greene, M. Olivier, *Physiol. Genomics* **2007**, *30*, 89.

^{1}H NMR

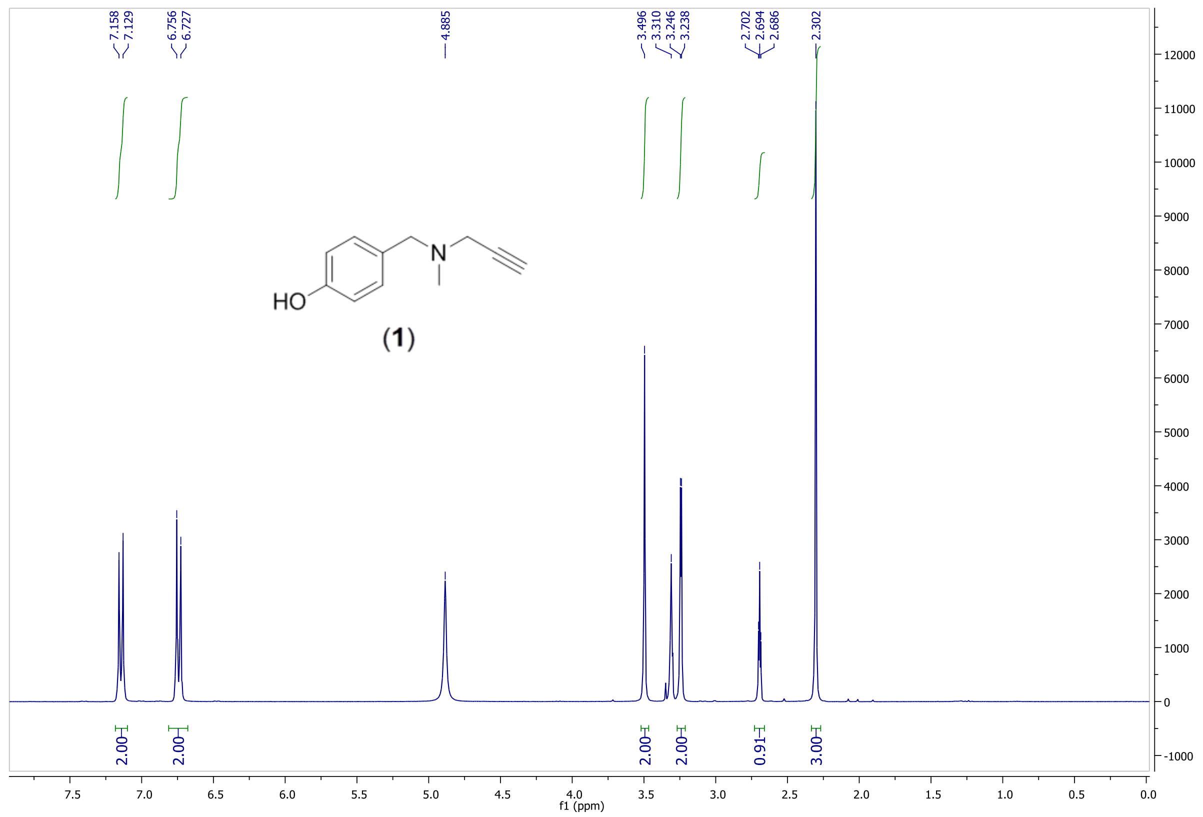

^{13}C NMR

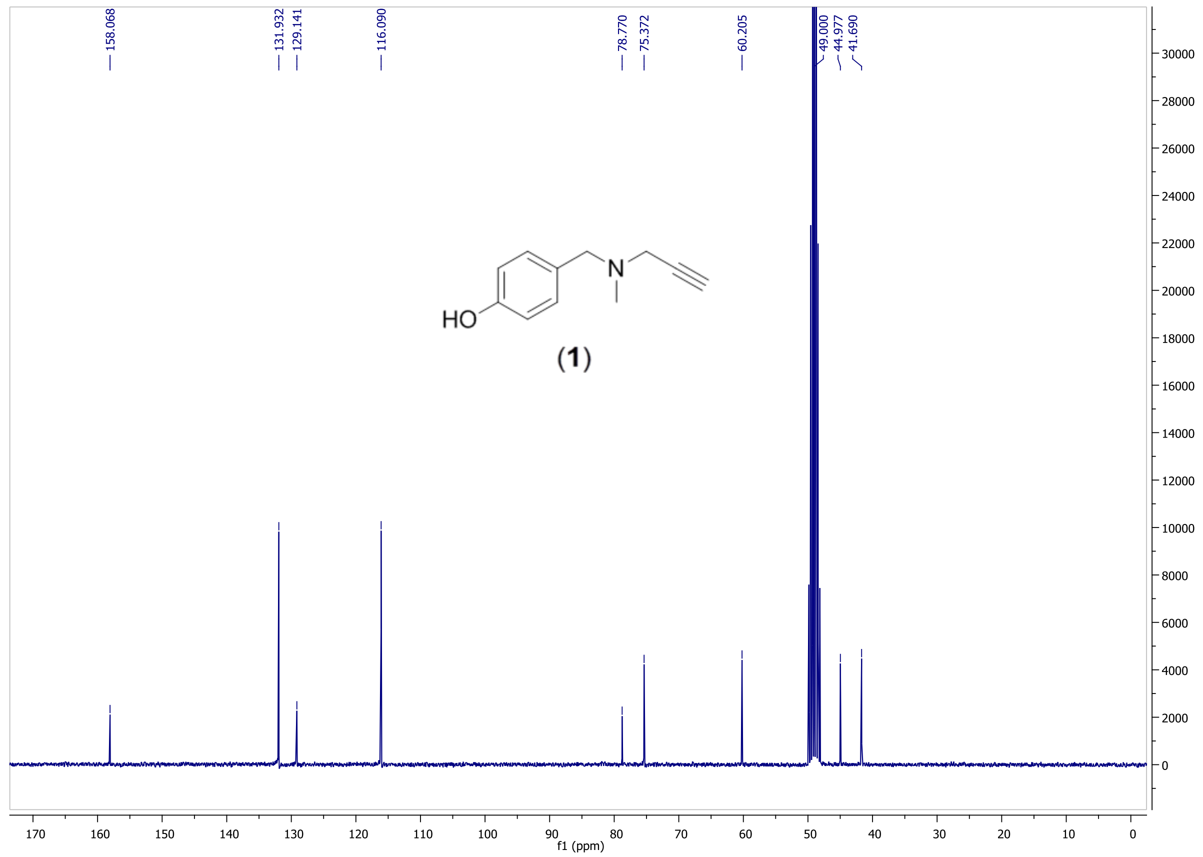

^{1}H NMR

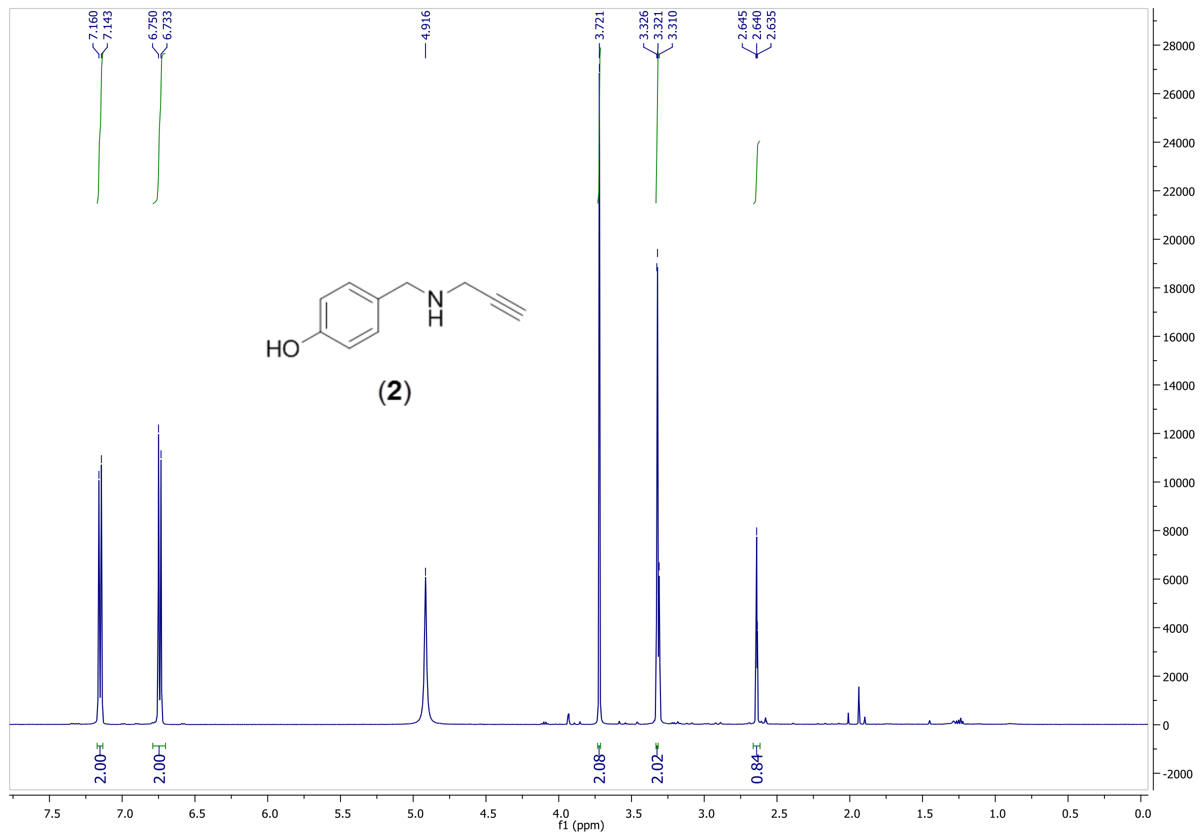

^{13}C NMR

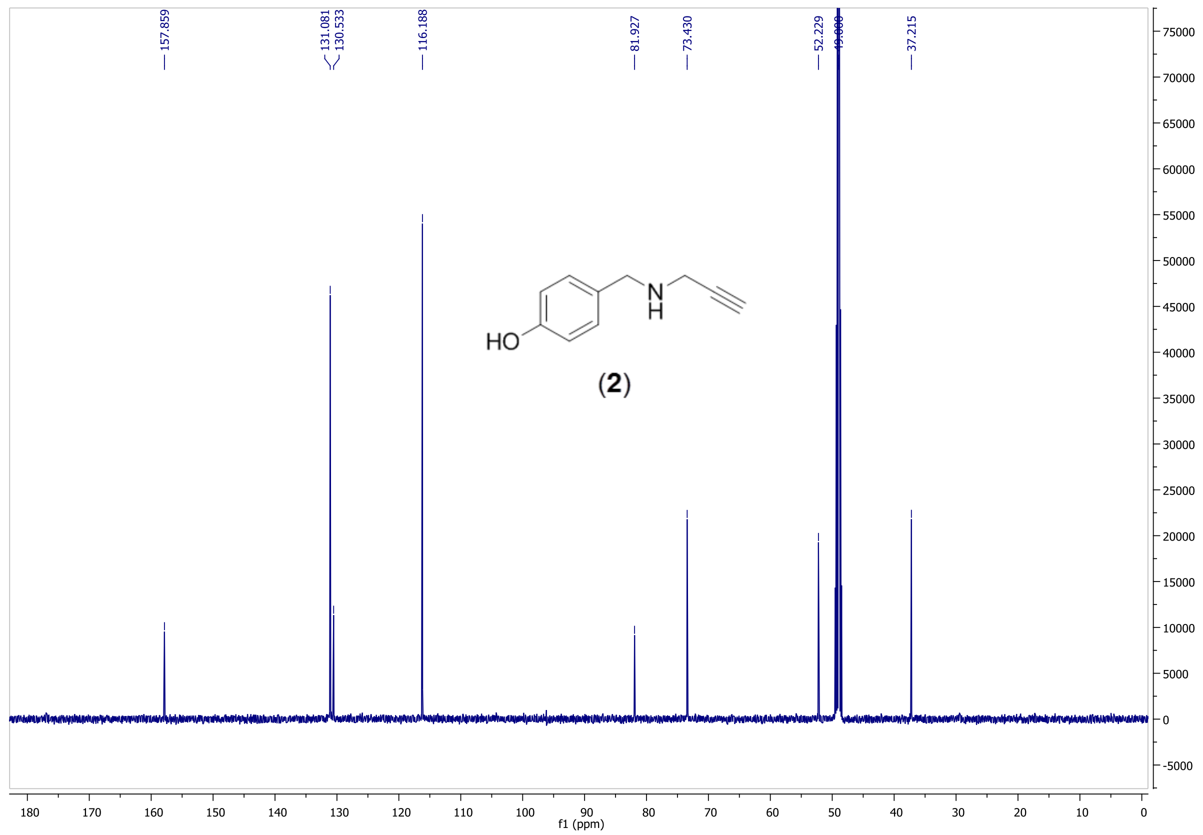

^{1}H NMR

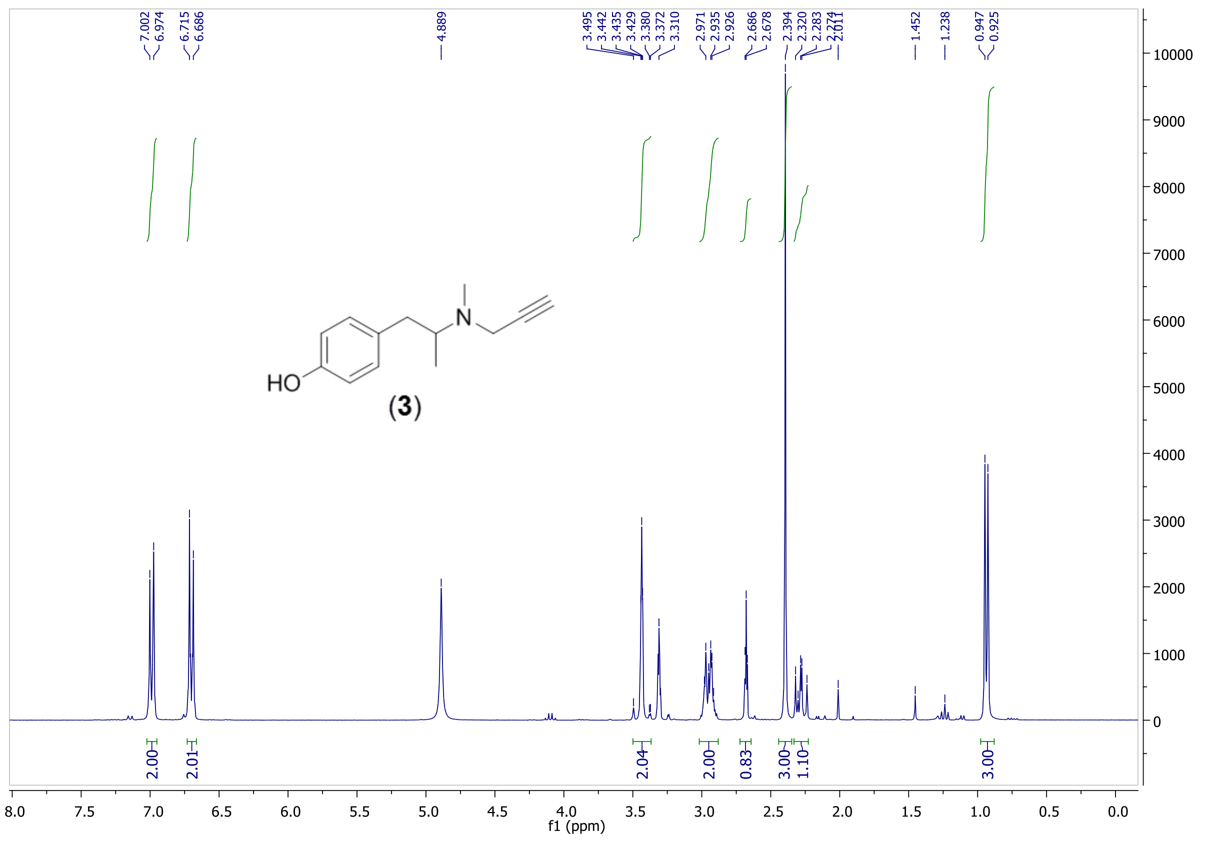

^{13}C NMR

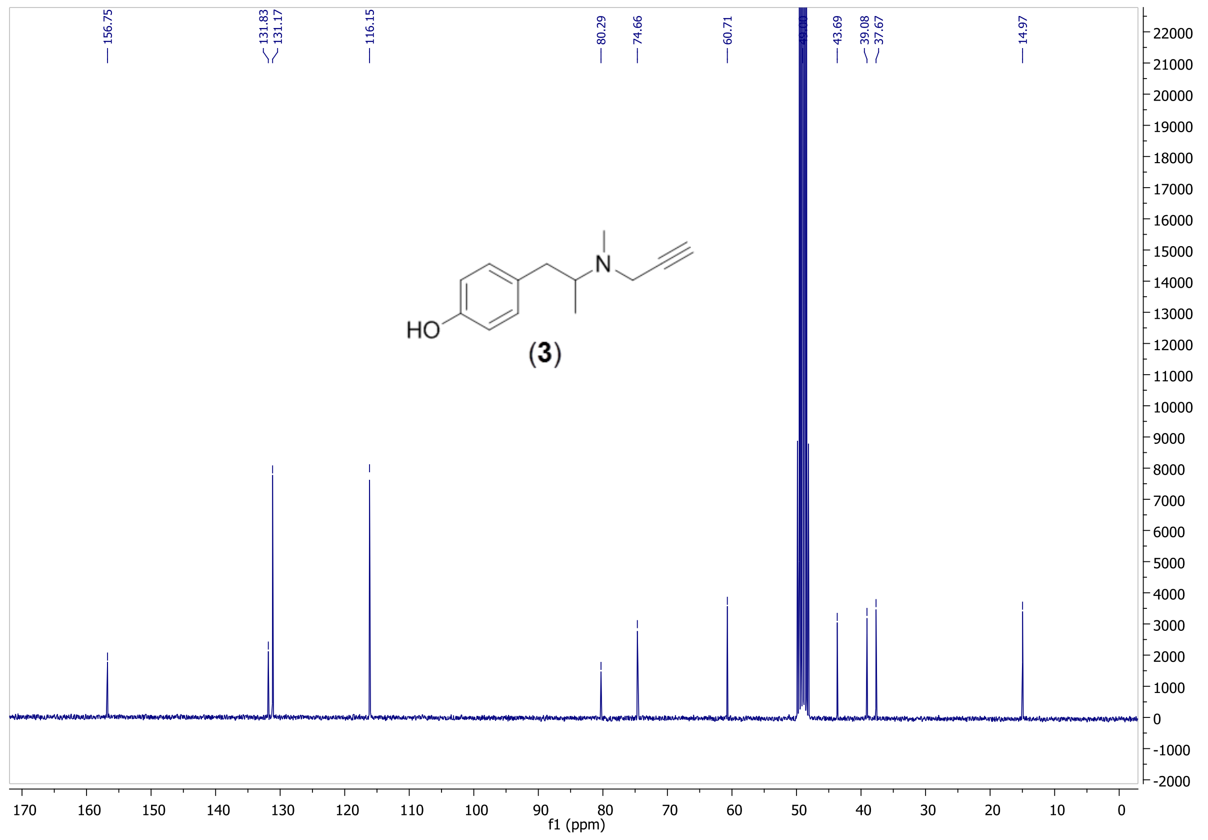

^{1}H NMR

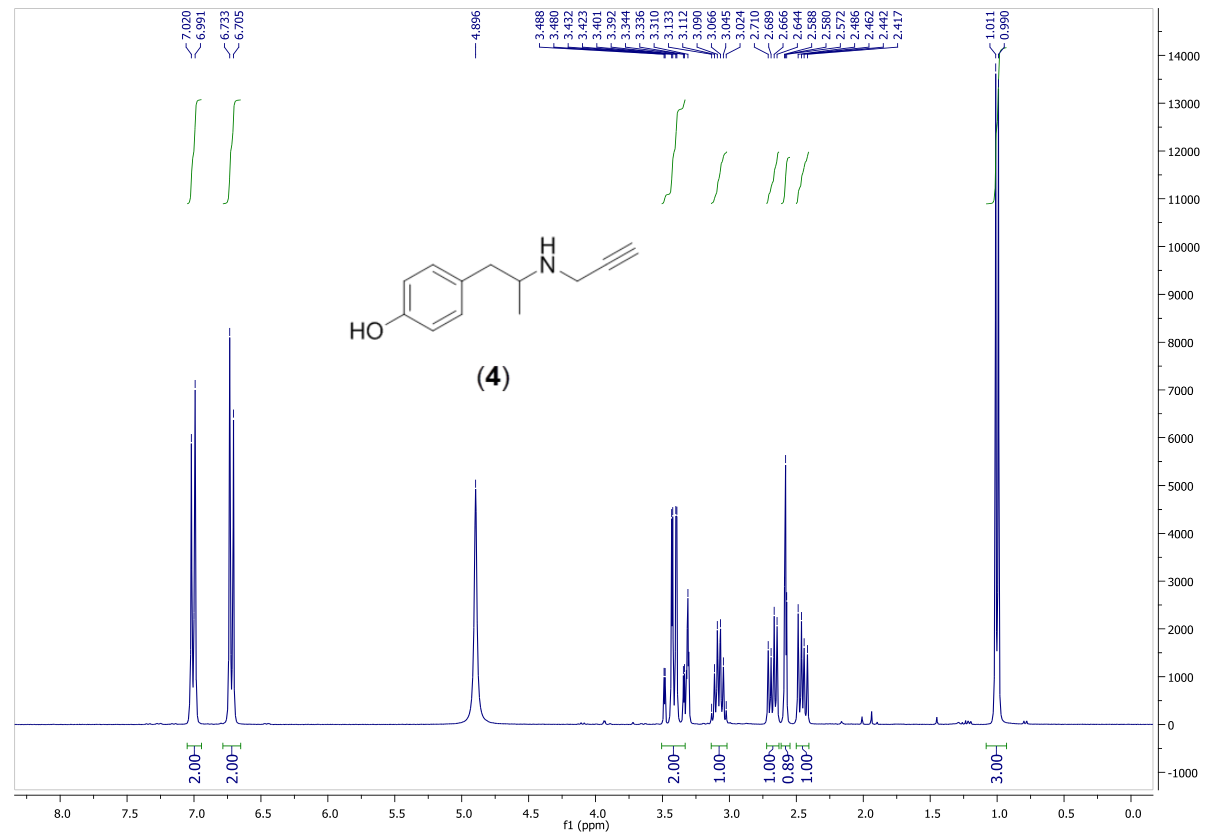

^{13}C NMR

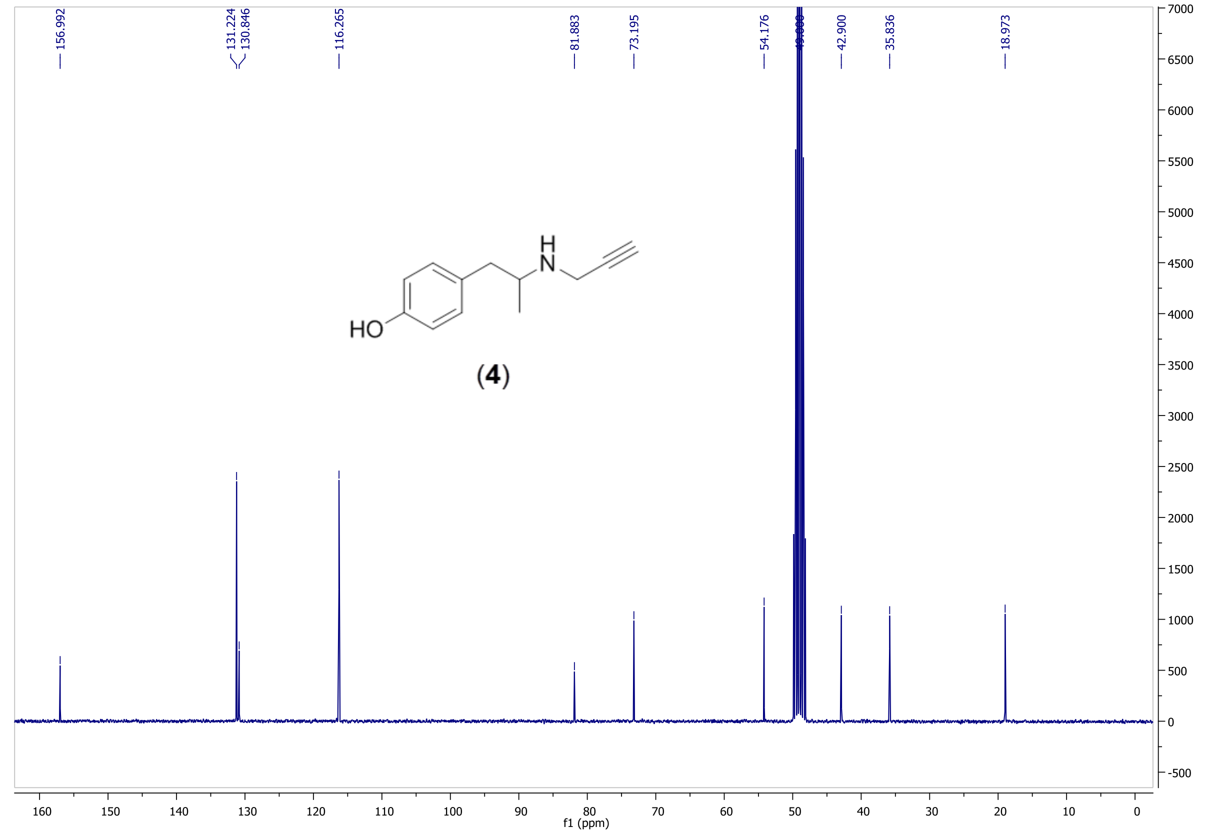

^{1}H NMR

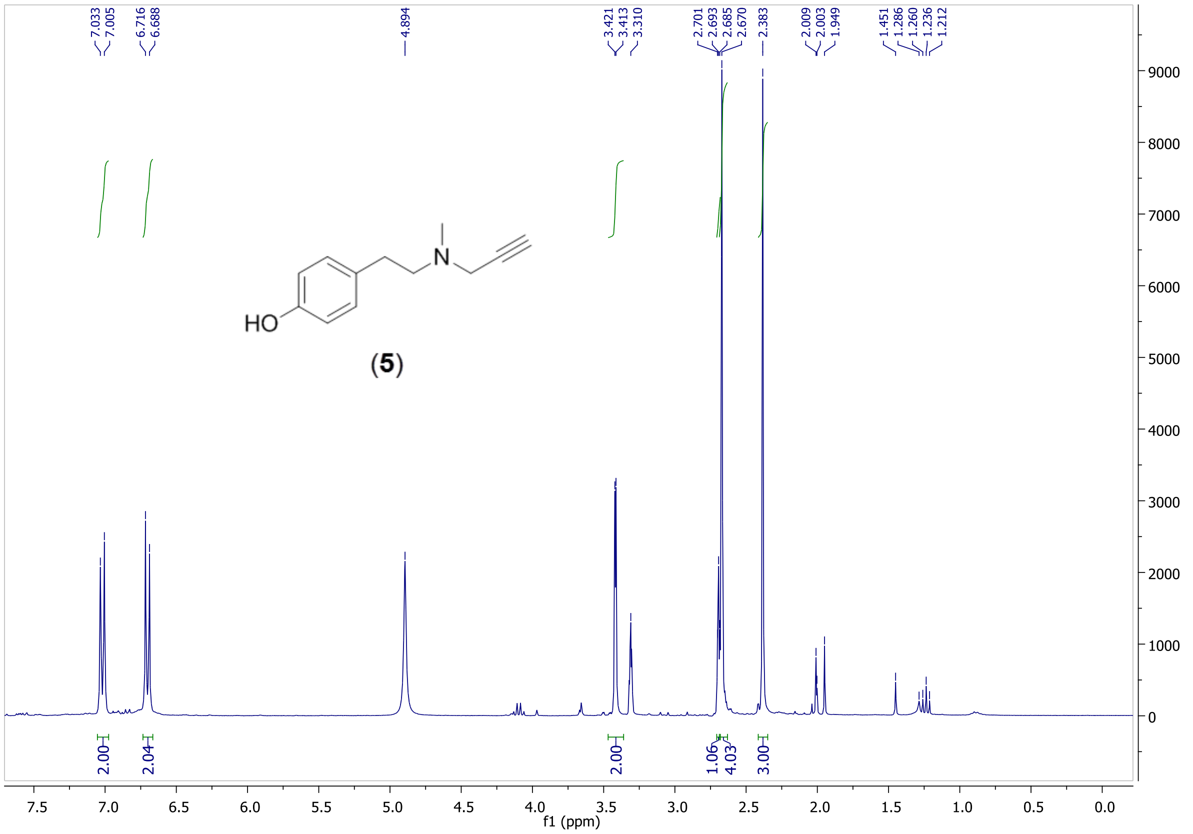

^{13}C NMR

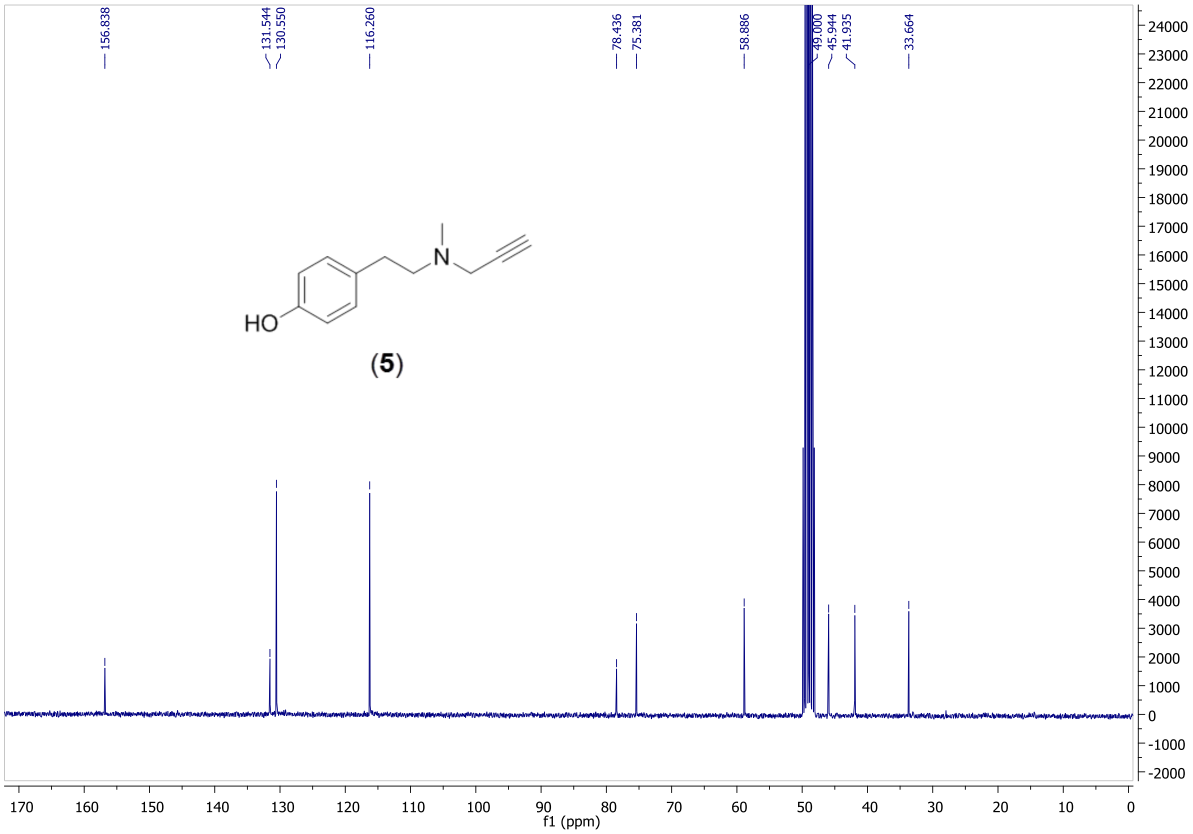

^{1}H NMR

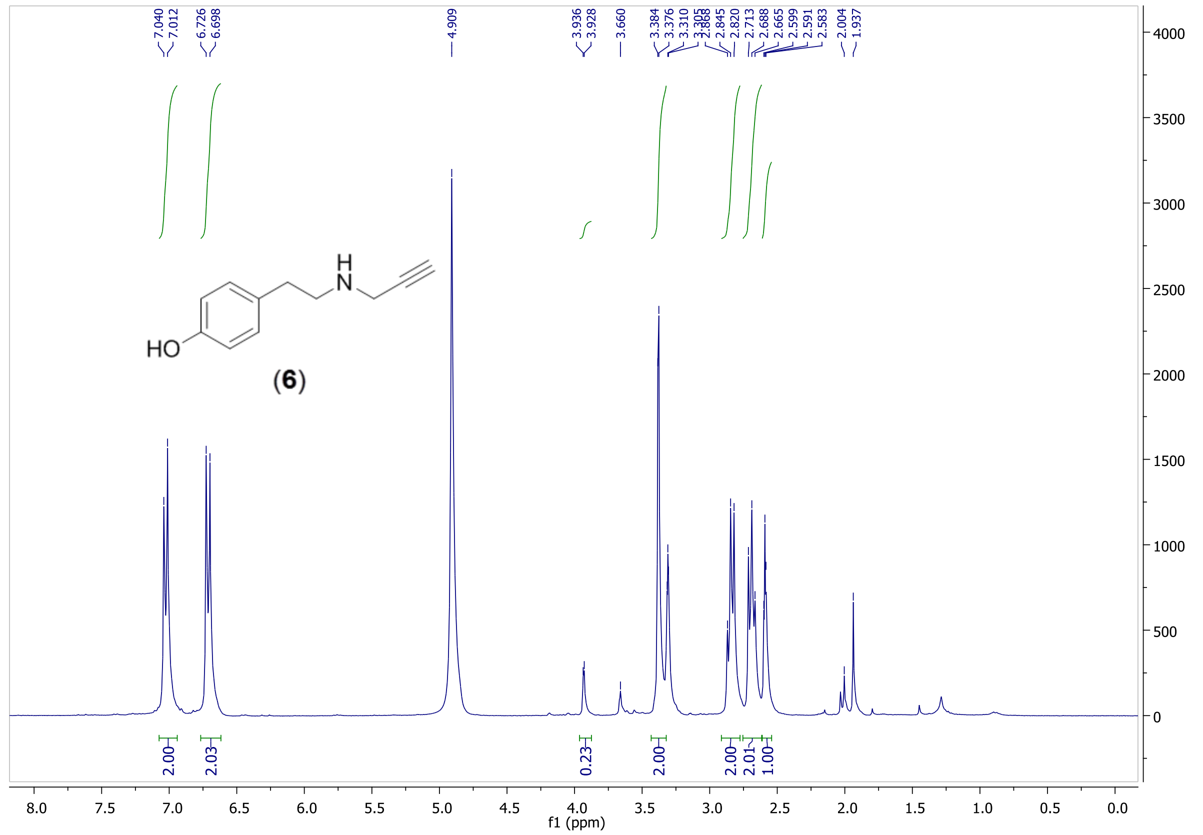

^{13}C NMR

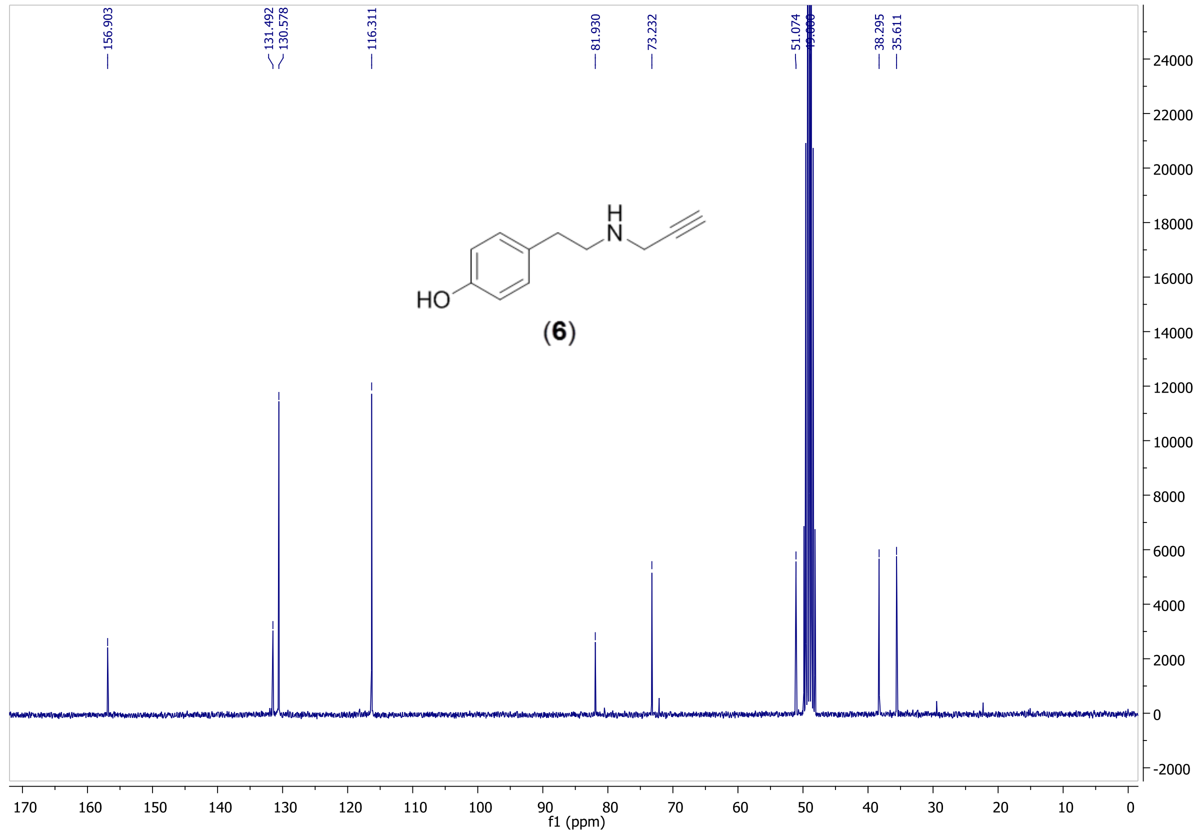

^{1}H NMR Probe **P1**

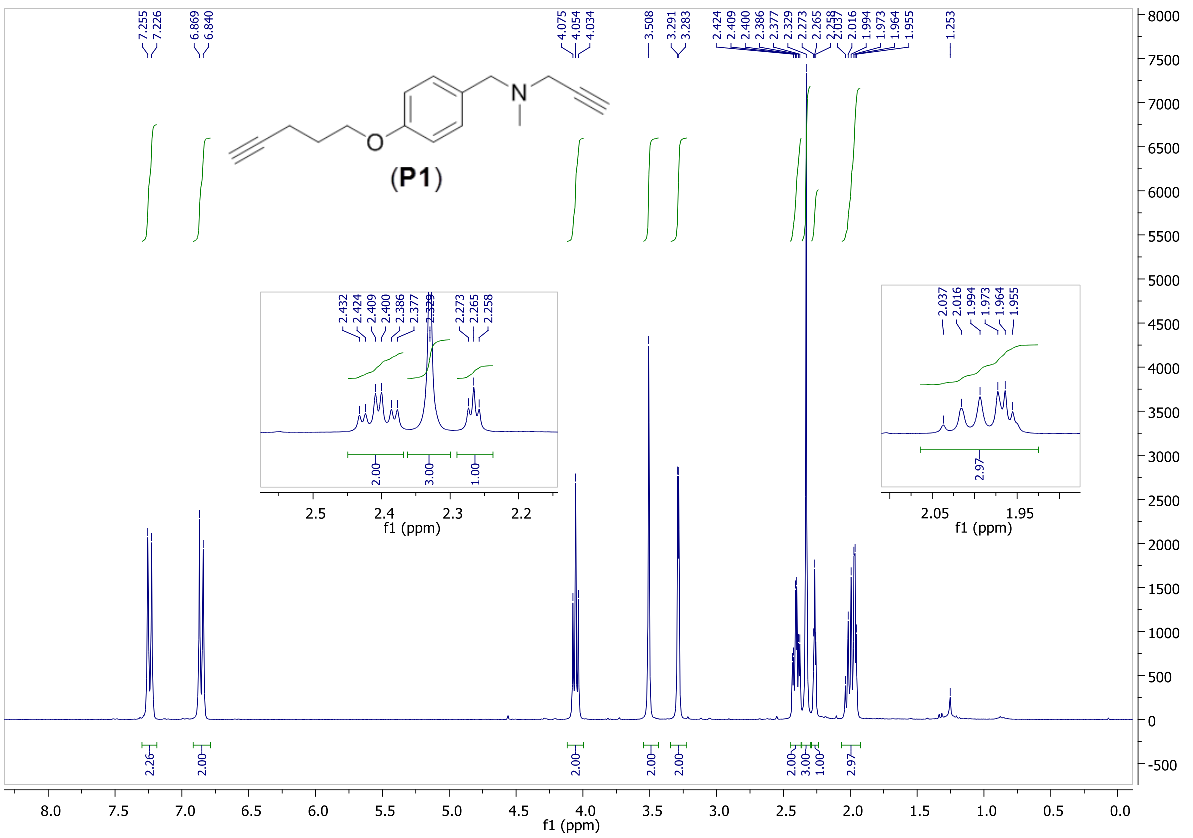

^{13}C NMR Probe **P1**

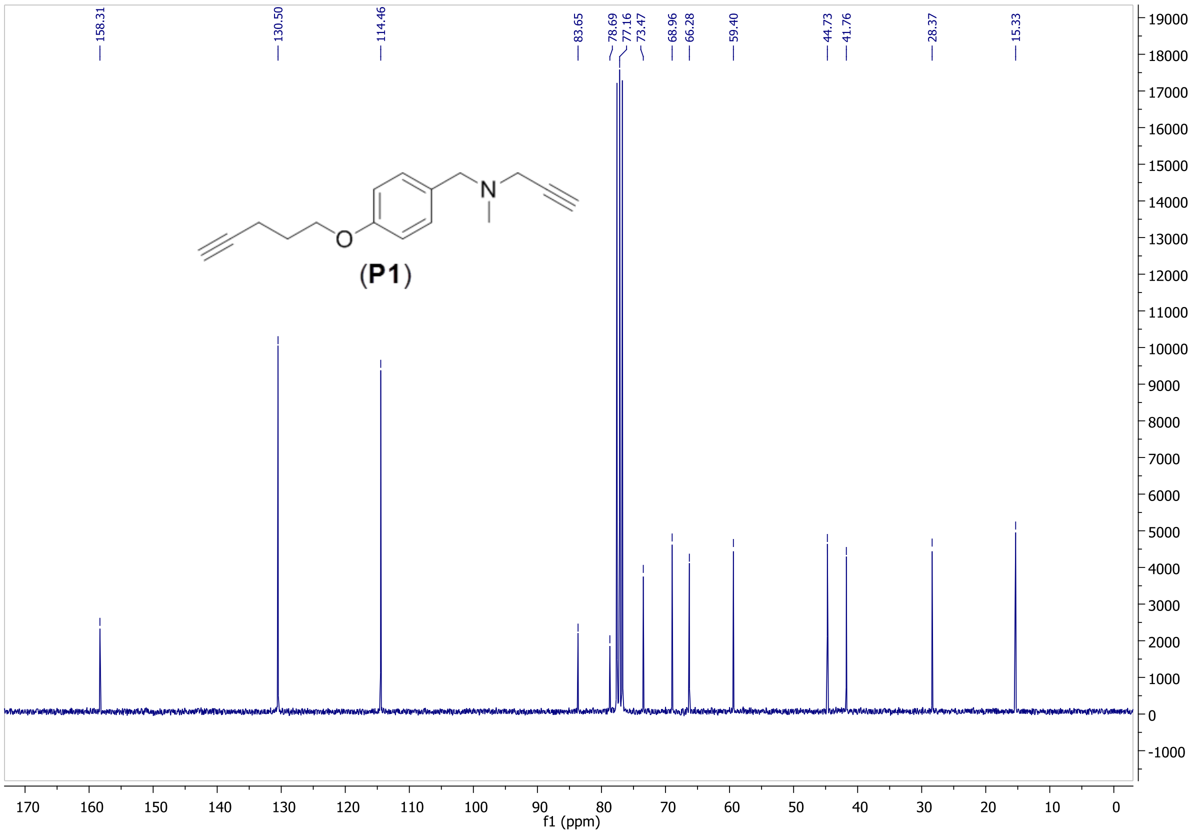

^{1}H NMR Probe **P2**

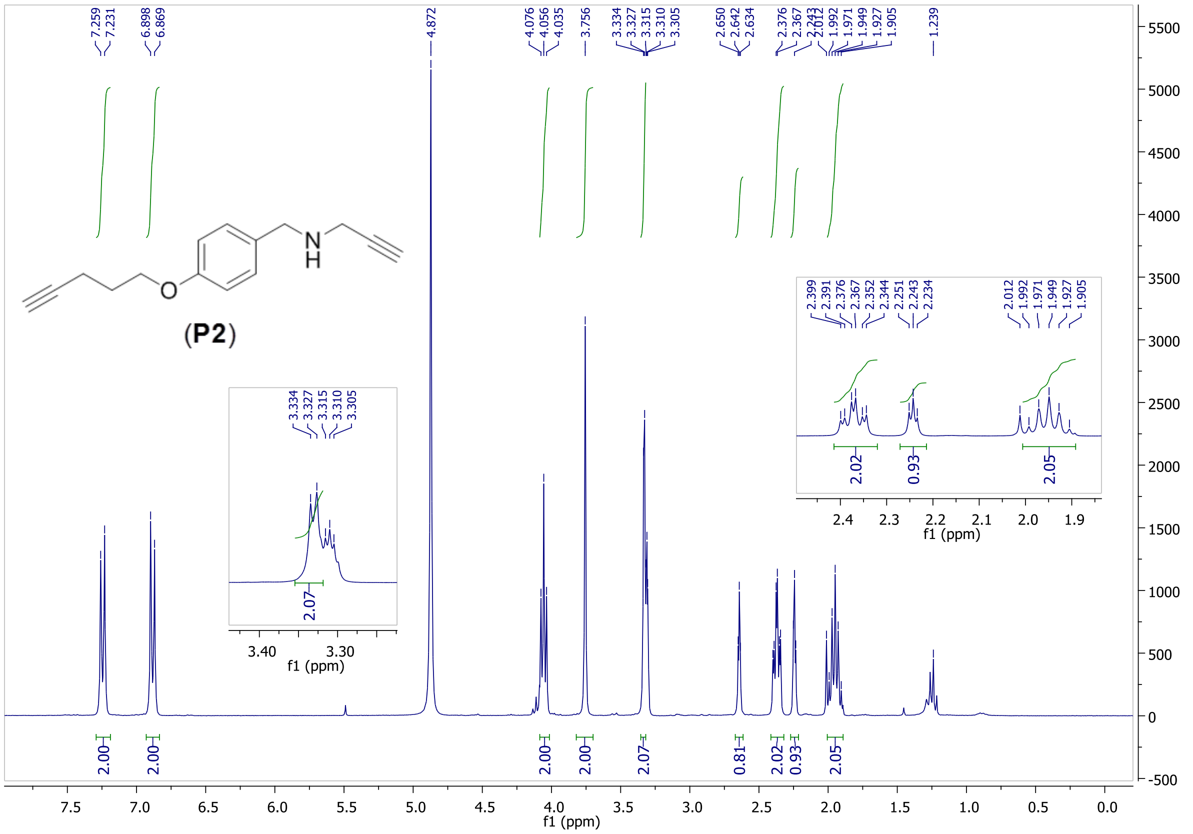

^{13}C NMR Probe **P2**

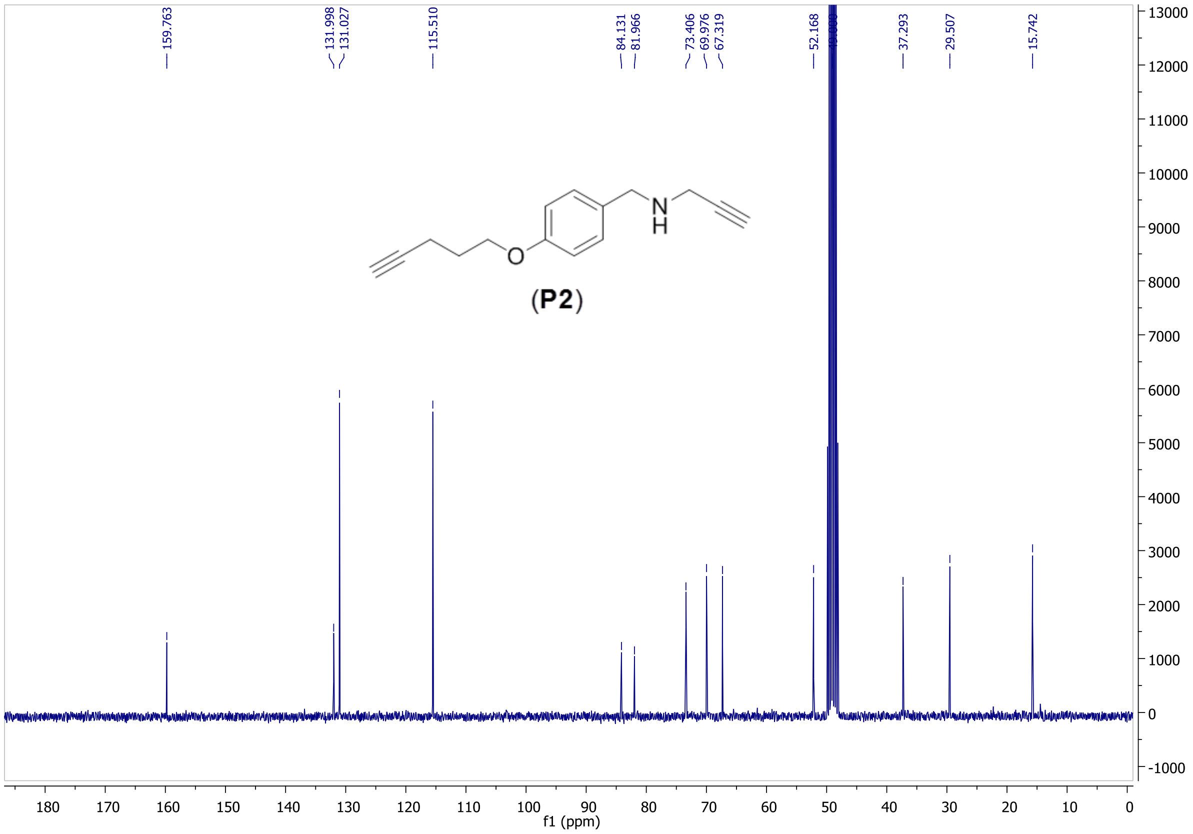

^{1}H NMR Probe **P3**

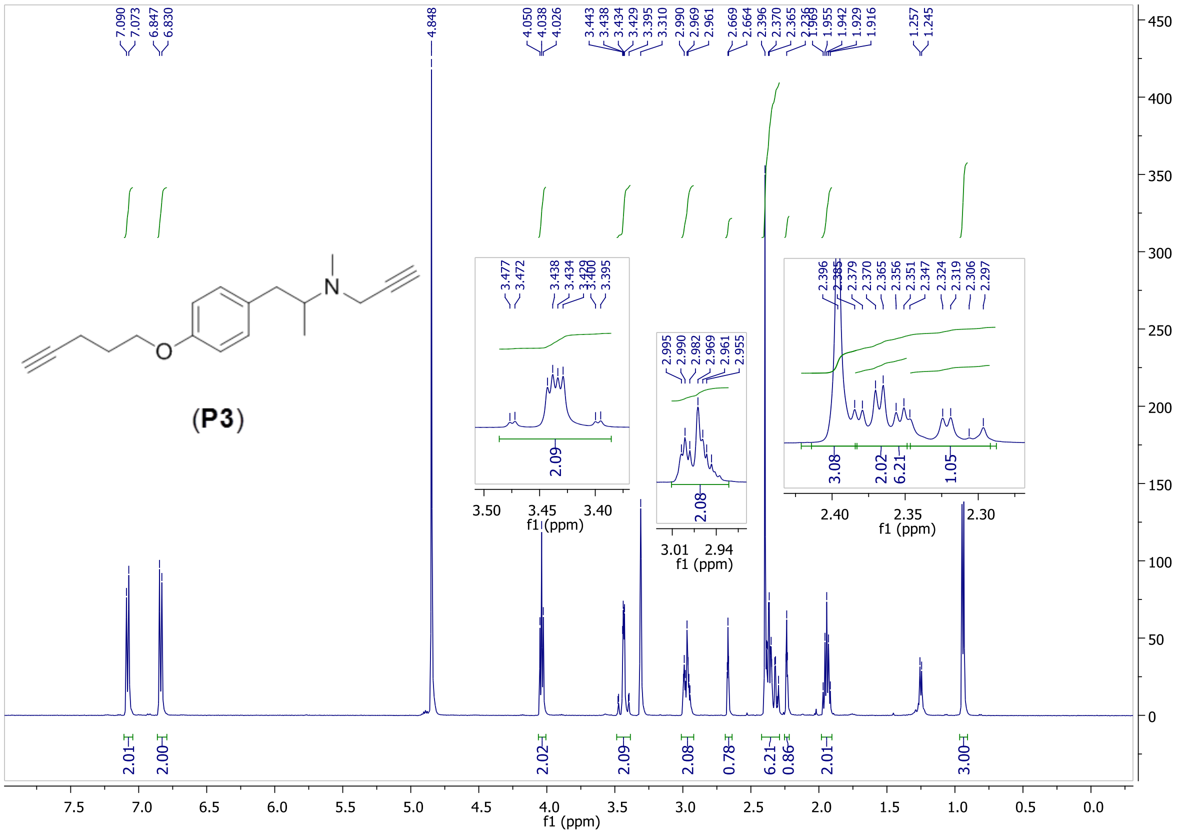

^{13}C NMR Probe **P3**

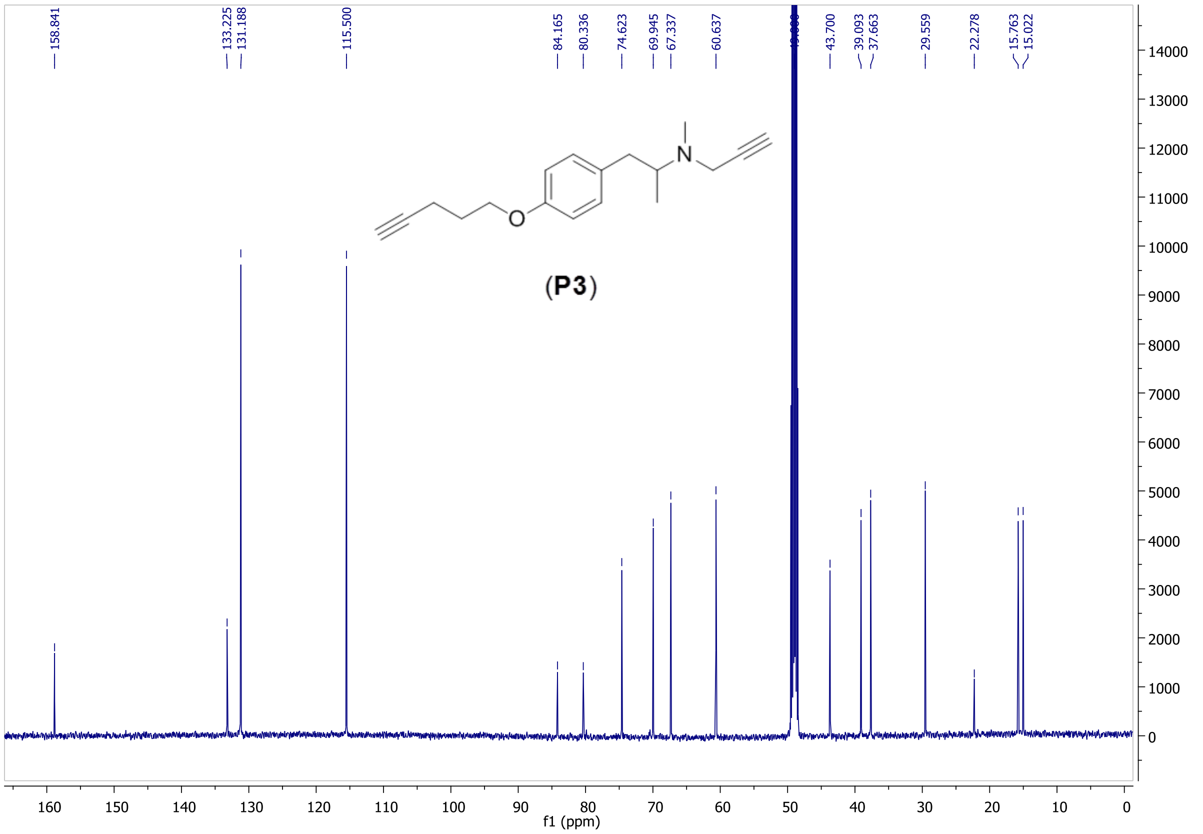

^{1}H NMR Probe **P4**

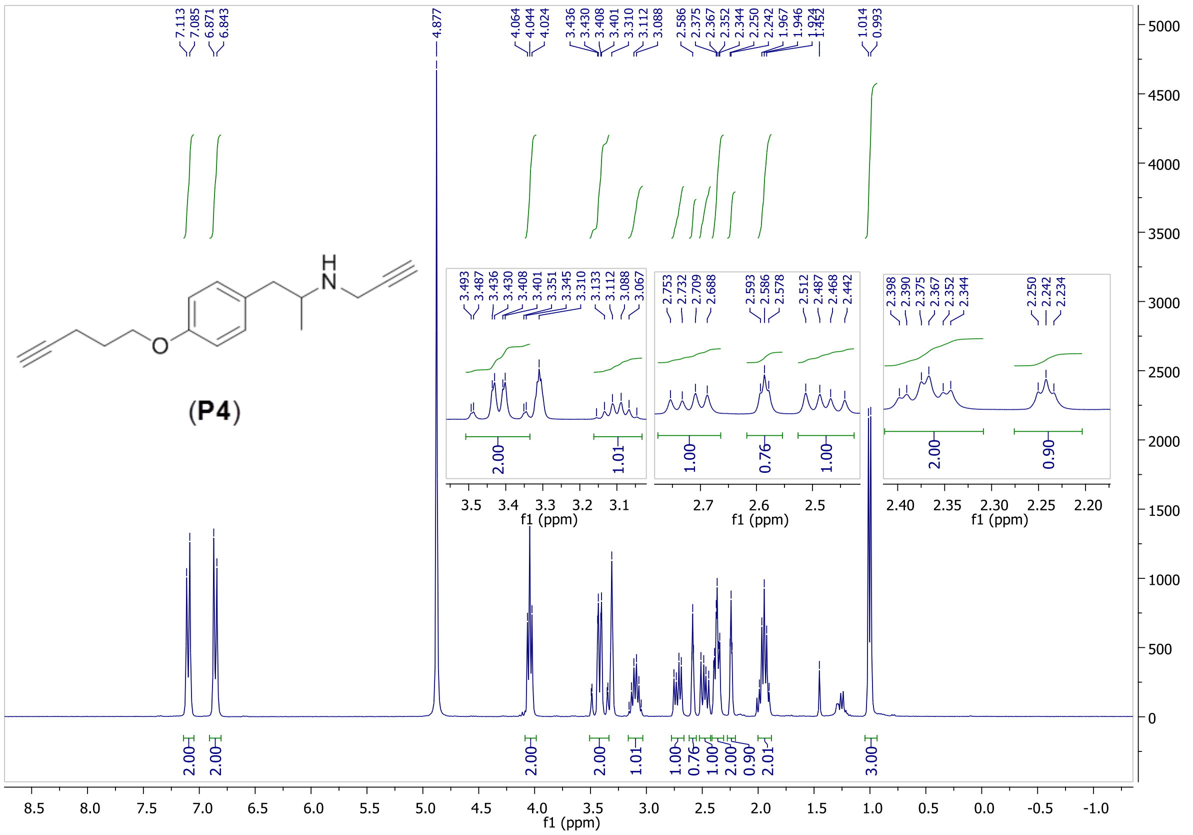

^{13}C NMR Probe **P4**

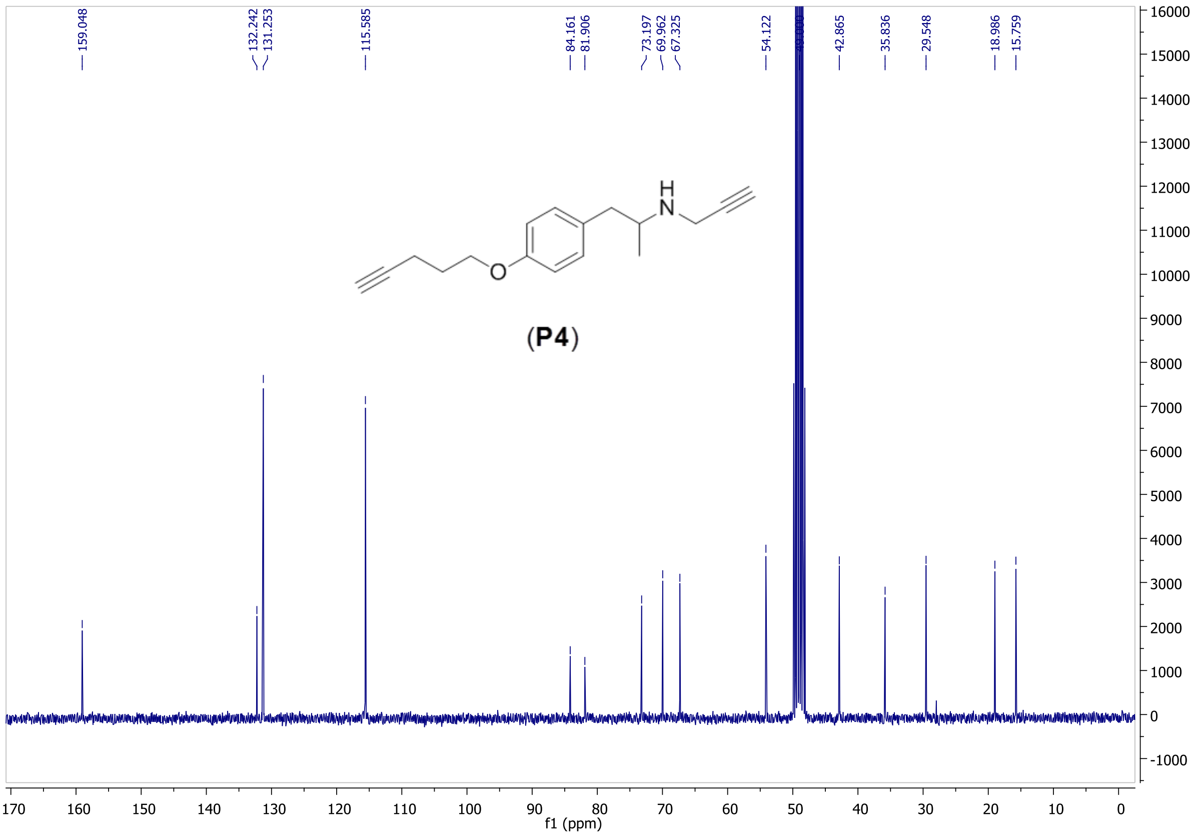

^{1}H NMR Probe **P5**

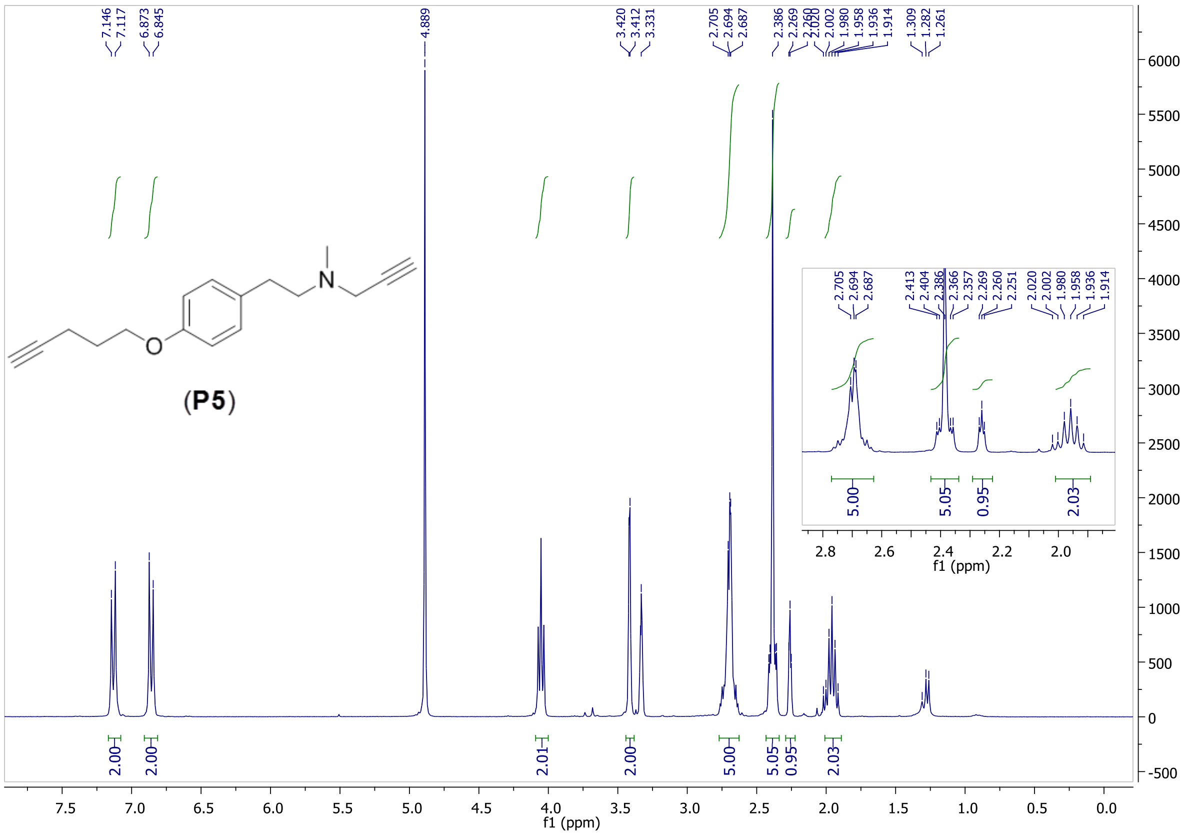

^{13}C NMR Probe **P5**

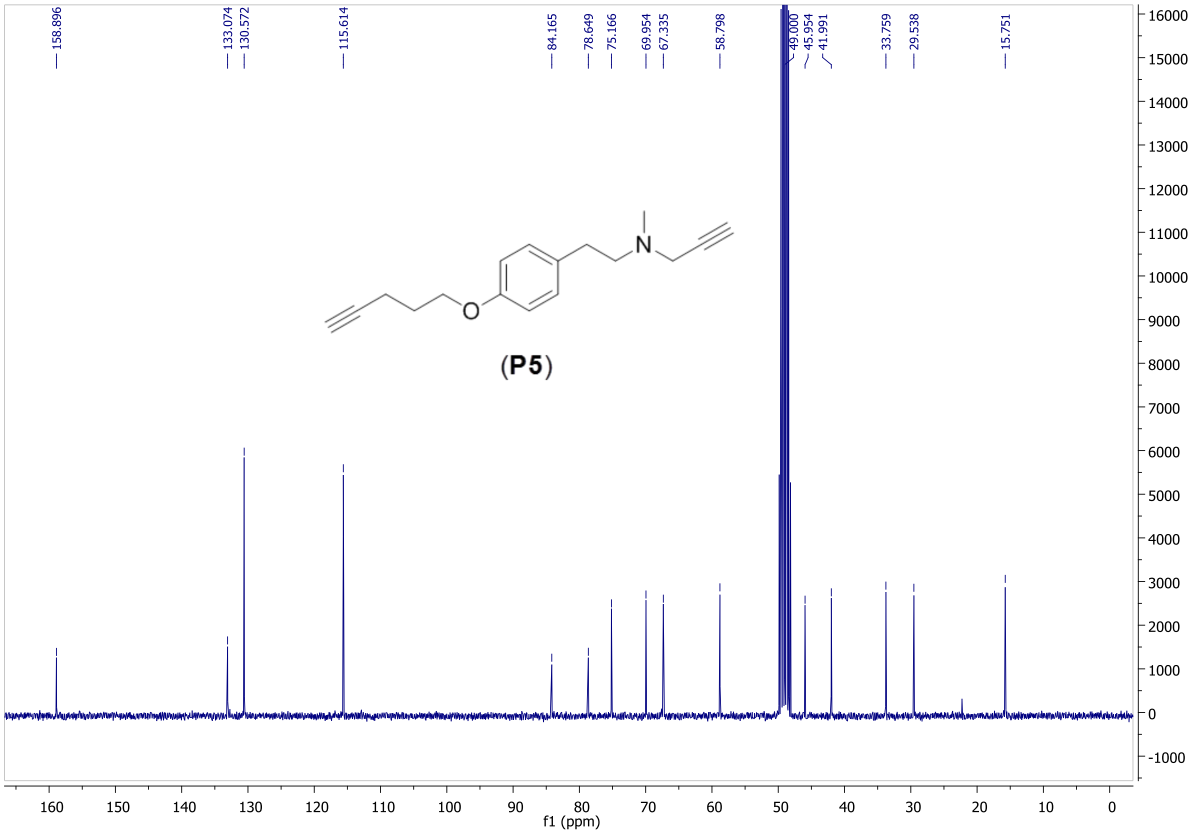

^{1}H NMR Probe **P6**

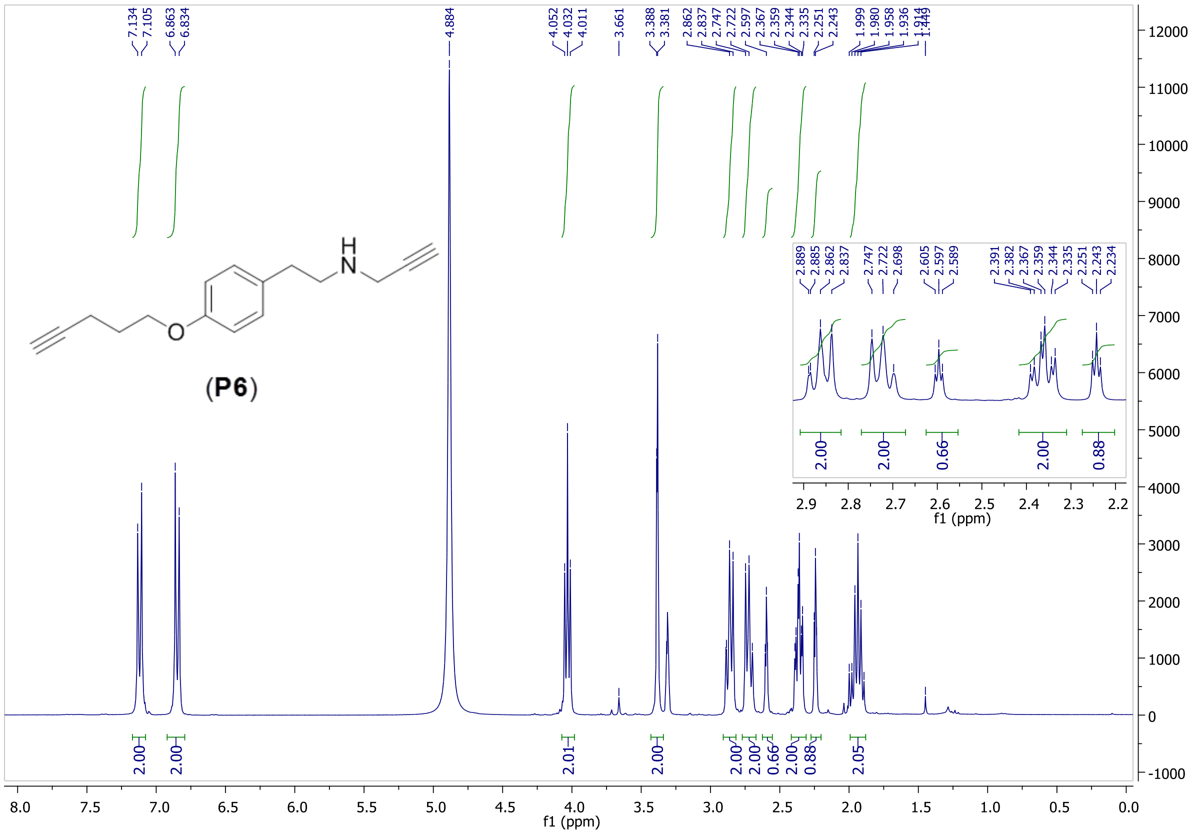

^{13}C NMR Probe **P6**

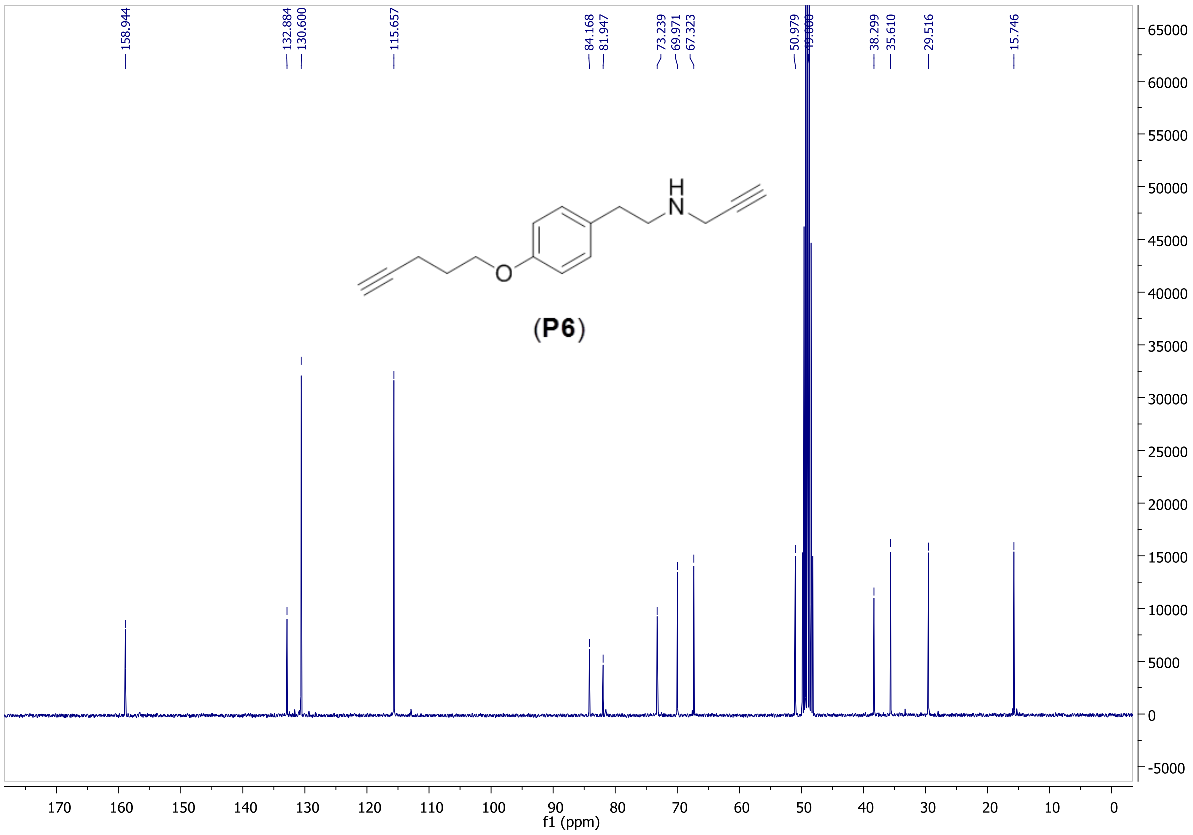

Supplement: Supplementary file 1 [file anie0051-7035-SD1.pdf]
